# Mechanisms of the Drug Penetration Enhancer Propylene Glycol Interacting with Skin Lipid Membranes

**DOI:** 10.1021/acs.jpcb.3c06784

**Published:** 2024-04-16

**Authors:** Jade Mistry, Rebecca Notman

**Affiliations:** Department of Chemistry, University of Warwick, Gibbet Hill Road, Coventry CV4 7AL, U.K.

## Abstract

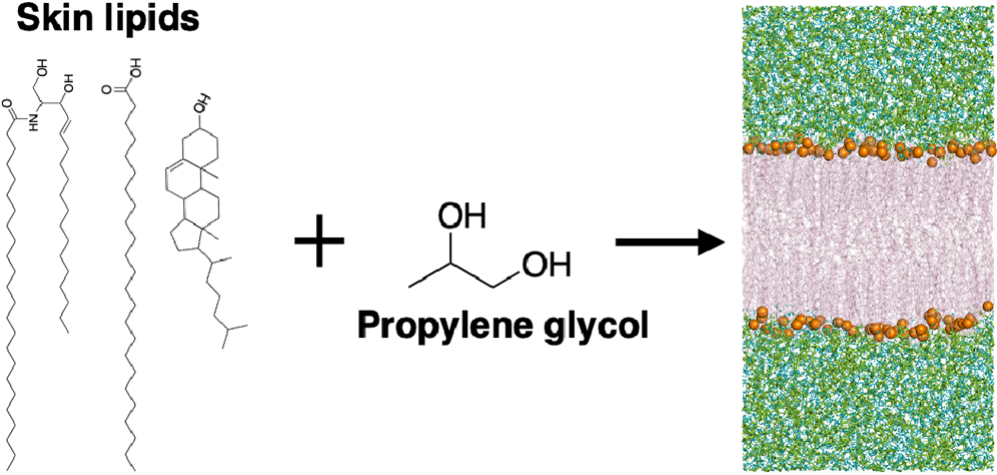

Very few drugs have the necessary physicochemical properties to cross the skin’s main permeability barrier, the stratum corneum (SC), in sufficient amounts. Propylene glycol (PG) is a chemical penetration enhancer that could be included in topical formulations in order to overcome the barrier properties of the skin and facilitate the transport of drugs across it. Experiments have demonstrated that PG increases the mobility and disorder of SC lipids and may extract cholesterol from the SC, but little is known about the molecular mechanisms of drug permeation enhancement by PG. In this work, we have performed molecular dynamics (MD) simulations to investigate the molecular-level effects of PG on the structure and properties of model SC lipid bilayers. The model bilayers were simulated in the presence of PG concentrations over the range of 0–100% w/w PG, using both an all-atom and a united atom force field. PG was found to localize in the hydrophilic headgroup regions at the bilayer interface, to occupy the lipid–water hydrogen-bonding sites, and to slightly increase lipid tail disorder in a concentration-dependent manner. We showed with MD simulation that PG enhances the permeation of small molecules such as water by interacting with the bilayer interface; the results of our study may be used to guide the design of formulations for transdermal drug delivery with enhanced skin permeation, as well as topical formulations and cosmetic products.

## 1 Introduction

Transdermal drug delivery (TDD) is a method of transporting drugs through skin directly into the bloodstream. It provides an attractive alternative to oral and intravenous delivery as it is easy to use, noninvasive, and allows for controlled delivery of drugs.^1^ TDD systems also benefit from reduced side effects and lower toxicity than oral drugs.^1^ However, a major limitation of TDD is that very few drugs have the necessary physicochemical properties to cross the skin’s main permeability barrier, the stratum corneum (SC), in sufficient amounts. One strategy to overcome this barrier is to use chemical penetration enhancers^2,3^ that interact with skin lipids to increase the permeability of the SC and facilitate the transport of drugs across the skin.

The SC is the outermost layer of the skin and consists of layers of corneocytes embedded in a multilamellar lipid matrix in a “bricks and mortar” arrangement.^4^ The lipid matrix serves as the only continuous pathway through the SC and comprises a heterogeneous mixture of ceramides, free fatty acids (FFAs), and cholesterol (CHOL) in a roughly equimolar ratio.^5^ The lipids form lamellar structures that are considered to exist as trilayers^4^ or stacked bilayers,^6^ though their precise molecular organization remains unknown. The lipid matrix also has two coexisting phases: a long periodicity phase that contains very long-chain ceramides and has a repeat distance of 13 nm and a short periodicity phase that contains shorter-chain ceramides and has a repeat distance of roughly 6 nm.^4,7,8^ At typical skin temperatures, the skin lipids are thought to exist primarily in gel or crystalline phases.^9^

Ceramides make up the main components of the lipid matrix by weight and play an important role in the barrier function of the SC. They consist of a sphingoid base and a fatty acid chain linked via an amide bond, and at least 22 different ceramide subclasses are thought to exist in human SC.^10^ Ceramides are often found in the hairpin conformation,^11,12^ where both chains point in the same direction, though the multilamellar arrangement of SC lipids makes an extended^11−13^ (or splayed) conformation, where the ceramide chains point in opposite directions, also possible. The FFAs of the SC typically have a chain length of 18 carbon atoms or more, with the two most abundant being lignoceric acid (24 carbons, FA24) and cerotic acid (26 carbons, FA26).^12^ The FFAs are often tightly packed and regulate the integrity of the SC barrier,^14^ whereas CHOL fluidizes the membrane.^15^

Chemical penetration enhancers are used in TDD to temporarily overcome the barrier properties of the SC and aid the transport of drugs across skin. Some common classes of penetration enhancers include sulfoxides, alcohols, glycols, fatty acids, and terpenes. Propylene glycol (PG) is a penetration enhancer^3,16,17^ that is widely used in transdermal drug patches^18−20^ and topical skin treatments.^21−24^ It can also act as a cosolvent^25,26^ to increase the thermodynamic activity of a drug and may be used alone or in combination with other penetration enhancers such as oleic acid.^27−29^

One of the main ways PG is thought to enhance the permeation of drug molecules through skin is by interacting with SC lipids. It is well documented in the literature that PG penetrates skin and partitions into the SC.^25,27,30−32^ Small-angle X-ray diffraction (SAXD) and small-angle X-ray scattering (SAXS) studies reveal that PG is able to intercalate into the lipid headgroup regions of the SC, increase the interfacial area per lipid (APL), and disrupt the lateral packing of lipids.^7,33^ Experiments have also demonstrated that PG increases the mobility of SC lipids^34^ and disorders the lipid bilayers.^31^ Furthermore, according to FTIR spectroscopy studies, PG may also extract lipids from the SC, providing a potential permeation pathway for drug molecules.^27,35,36^

Despite numerous experimental studies and its widespread use, the penetration-enhancing effects of PG at the molecular level are still poorly understood. Molecular dynamics (MD) simulations may be used to gain a molecular-level understanding of the penetration-enhancing mechanisms of PG. MD simulation studies of phospholipid bilayers in the presence of PG have been performed.^37−39^ Phospholipids are an important class of lipids that are found in many biological cell membranes, though they are virtually absent from the SC.^12^ In simulations of 1,2-dioleoyl-*sn*-glycero-3-phosphocholine (DOPC) bilayers in the liquid crystalline phase with PG, Hughes et al.^37^ found that the accumulation of PG at the DOPC headgroups caused the membrane to expand laterally. The presence of PG at the interface also led to a decrease in the number of DOPC–water hydrogen bonds (H-bonds), resulting in partial dehydration of the lipid headgroups. PG was able to penetrate into the hydrocarbon region of the membrane and diffuse across the DOPC bilayer. PG induced pore formation at concentrations of 15 and 25 mol % PG. It was also found to thin the membrane and disorder the lipid acyl tails, with higher concentrations of PG causing a greater disordering effect. Similar findings were reported by Malajczuk et al.^38^ for MD simulations of PG with pure 1,2-dipalmitoyl-*sn*-glycero-3-phosphatidylcholine (DPPC) bilayers in the liquid crystalline phase. PG showed a tendency to aggregate in the phosphate headgroup regions of the DPPC bilayers and caused lateral expansion of the membrane, as well as disordering the lipid tails and decreasing the bilayer thickness.

**Figure 1:**
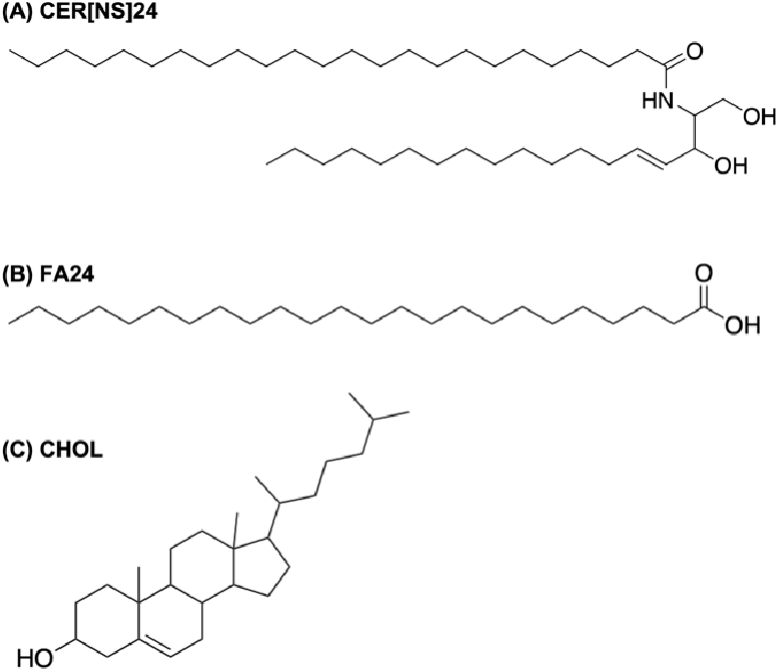
Structure of the lipids used in the model SC bilayers simulated in this work. CER[NS]24 consists of a sphingoid base (S) connected to a non-hydroxy fatty acid (N) with a chain length of 24 carbon atoms by an amide bond.

In the present study, we have performed MD simulations of model SC lipid bilayers comprising a mixture of CER[NS]24, FA24, and CHOL in a 1:1:1 molar ratio in the presence of 0, 20, 40, 60, 80, and 100% w/w PG, using an all-atom (AA) and a united atom (UA) force field (the molecular structure of the lipids is given in Figure 1). The work aimed to investigate how PG interacts with the SC lipids at the molecular level and reveal the effects of PG on SC-lipid bilayer properties. To the best of our knowledge, these are the first reported simulations of SC lipids in the presence of PG. The work represents an important step toward gaining an understanding of how PG enhances the penetration of drugs across skin.

## 2 Methods

### 2.1 Propylene Glycol Models

**Figure 2:**
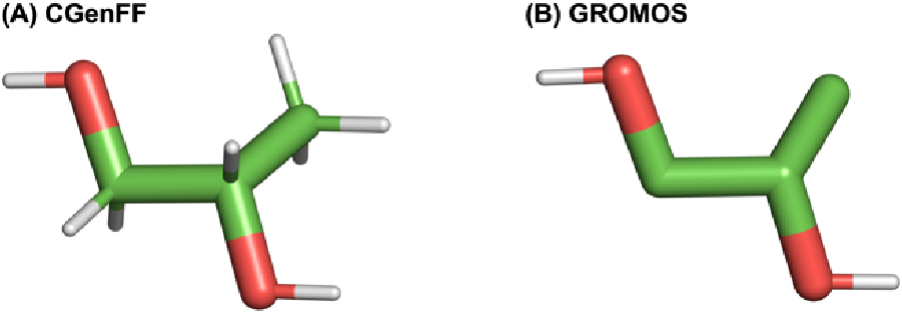
(A) All-atom and (B) united atom models of PG used in this work.

The CHARMM general force field (CGenFF) parameters for the AA model of PG were obtained from the CGenFF server version 4.5^40,41^ using a mol2 input file generated by Avogadro.^42^ UA parameters for PG were obtained using the Automated Topology Builder (ATB),^43^ along with the corresponding GROMOS 54A7 force field^44^ files. The structures of the AA and UA PG models used in this work are shown in Figure 2. GROMACS-compatible topology (.itp) files for both models are provided as Supporting Information. To validate the PG models, simulations of pure PG and selected PG/water mixtures were carried out, and the results were compared to experimental data and electronic structure calculations as described below.

#### 2.1.1 Simulations of Pure PG

All simulations were performed using GROMACS 2020.4.^45^ The following simulation protocol was used for both the AA and UA systems. 250 PG molecules were placed inside a 5 × 5 × 5 nm^3^ box and minimized for a maximum of 50000 steps using the steepest descent algorithm. The systems then underwent a 50 ps *NVT* equilibration followed by a 1 ns *NPT* equilibration. Finally, a 50 ns production run was performed in the *NPT* ensemble at 298.15 K and 1 bar pressure. The temperature was controlled using the Nosé–Hoover thermostat^46^ with a time constant of 1 ps, and the pressure was controlled isotropically using the Parrinello–Rahman barostat^47^ with a time constant of 5 ps and compressibility of 4.5 × 10^–5^ bar^–1^. All bonds were constrained using the LINCS algorithm.^48^ Electrostatic interactions were calculated using the particle mesh Ewald (PME) method.^49^ The cutoff distance for the van der Waals interactions and the short-range part of the electrostatics was 1.2 nm.

Partition coefficients describe the ratio of concentrations of a solute in a system containing two immiscible solvents:
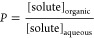
1

The logarithm of the octanol/water partition coefficient (log *P*) is often used in the pharmaceutical industry as a measure of a drug’s lipophilicity and to estimate how a drug molecule will partition into a lipid environment (represented by the octanol phase). More negative values of log *P* indicate that a molecule is more hydrophilic, while more positive values indicate that a molecule is more hydrophobic. A value of 0 indicates the molecule partitions into the organic and aqueous phases in a 1:1 ratio. The log *P* can be calculated from hydration and solvation free energies using MD simulations. Since log *P* is proportional to the transfer free energy of a solute moving from the aqueous phase to the organic phase, it can be estimated directly from the following equation:^50^
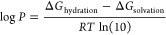
2where Δ*G*_hydration_ is the free energy change of removing one molecule of PG from water, and Δ*G*_solvation_ is the free energy change of removing one molecule of PG from octanol.

Hydration and solvation free energies for both the AA and UA models of PG were calculated via alchemical free energy perturbation (FEP) simulations. Data were analyzed via the GROMACS “bar” module, which uses the Bennett Acceptance Ratio (BAR) method^51^ to calculate free energy differences.

The following simulation parameters were used for both the AA and UA systems. One PG molecule was added to a 5 × 5 × 5 nm^3^ box and solvated with 376 octanol molecules or 4049 water molecules for the AA systems and 364 octanol or 4051 water molecules for the UA systems. The solvated systems were energy minimized using the steepest descent algorithm for a maximum of 50000 steps and then underwent *NVT* and *NPT* equilibration for 500 ps and 1 ns, respectively. Production runs were carried out in the *NPT* ensemble for 30 ns, which was sufficient to converge the calculated values of Δ*G*_hydration_ and Δ*G*_solvation_ (see Section S1.1).

Production run simulations were performed in the *NPT* ensemble at 298.15 K and 1 bar pressure. The temperature was controlled by Langevin dynamics, with a time constant of 0.5 ps. The pressure was controlled isotropically by the Parrinello–Rahman barostat with a time constant of 2 ps and compressibility of 4.5 × 10^–5^ bar^–1^. Bonds involving hydrogen atoms were constrained using the LINCS algorithm. Electrostatic interactions were calculated using the PME method. The cutoff distance for the van der Waals interactions and the short-range part of the electrostatics was 1.2 nm.

The last frame of the *NPT* equilibration simulation was used as the starting configuration for the windowed alchemical free energy simulations in the *NPT* ensemble. According to the protocol of Fan et al.,^52^ Coulomb interactions were switched off over seven windows for octanol simulations (coupling parameter λ_Coul_ ∈ {0, 0.125, 0.25, 0.375, 0.5, 0.75, 1}) and five windows for water simulations (coupling parameter λ_Coul_ ∈ {0, 0.25, 0.5, 0.75, 1}), while the van der Waals interactions were maintained (λ_vdW_ = 0). The van der Waals interactions were then switched off over 16 windows (λ_Coul_ = 1 and λ_vdW_ ∈ {0, 0.05, 0.1, 0.2, 0.3, 0.4, 0.5, 0.6, 0.65, 0.7, 0.75, 0.8, 0.85, 0.9, 0.95,1}).

The GROMACS “bar” module was used to obtain the Δ*G*_solvation_ of PG in octanol and water. These solvation free energies were then used to calculate the log *P*, according to eq 2.

Quantum mechanical calculations using ORCA 5.0 software^53^ were carried out to obtain the energy profile for the O–C–C–O dihedral angle of PG. First, the geometry of PG was optimized at the RHF/def2-TZVP level. Then, a relaxed scan of the O–C–C–O dihedral from 0 to 360° was performed at the MP2/def2-TZVP level, in steps of 10°.

### 2.2 Model Skin Lipid Bilayers

The AA and UA model SC bilayers used in this work were composed of CER[NS]24, FA24, and CHOL in an equimolar ratio. CER[NS]24 was selected as the main ceramide component since it is one of the most abundant and most studied ceramides, which enables comparison to extensive previous MD simulation work^54−58^ using this lipid bilayer configuration.

### 2.3 All-Atom Model Bilayer

The CHARMM36 force field^59,60^ was used to model the AA lipids, with water molecules described by the TIP3P water model.^61^ The CHARMM-GUI membrane builder^62,63^ was used to generate the initial configuration of the AA lipid bilayer (referred to as the “CHARMM” bilayer henceforth), which was composed of a random mixture of CER[NS]24, FA24, and CHOL in a 1:1:1 ratio. The bilayer contained 288 lipids in total and was solvated with water molecules at a 1:30 lipid:water ratio. Three independent replicas of the system with different starting positions of the lipids were generated by the CHARMM-GUI membrane builder.

Equilibration run protocols were provided by the CHARMM-GUI. Following energy minimization via the steepest descent algorithm, a series of short (125–500 ps) *NVT* and *NPT* equilibration runs were performed where the position restraints on the lipids were gradually turned off. Production run simulations were then carried out in the *NPT* ensemble for 500 ns, with a time step of 2 fs. The temperature of the lipids and water was controlled separately by the Nosé–Hoover thermostat at 305 K (a reasonable estimate of skin temperature), with a time constant of 1 ps. The pressure was controlled semi-isotropically using the Parrinello–Rahman barostat, with a time constant of 5 ps and compressibility of 4.5 × 10^–5^ bar^–1^. Bonds involving hydrogen were constrained using the LINCS algorithm. Electrostatic and van der Waals interactions were calculated using the PME method with a short-range cutoff distance of 1.2 nm and a force-switch from 1 to 1.2 nm for van der Waals interactions. Periodic boundary conditions (PBCs) were applied in the *x*, *y*, and *z* directions.

### 2.4 United Atom Model Bilayer

The UA model bilayer was the same one used previously by Del Regno and Notman^55^ and was composed of CER[NS]24, FA24, and CHOL in a 1:1:1 ratio. The interaction potentials used to describe the CER[NS]24 and FA24 tails were taken from Notman et al.,^64^ which was based on the UA force field developed by Berger et al.,^65^ with the partial charges given in Das et al.^56^ The polar parts of FA24 and CHOL were described using the force field of Höltje et al.^66^ Water was described by the SPC model.^67^ The bilayer contained 180 lipids in total and was solvated with water molecules at a 1:30 lipid:water ratio. Two more independent replicas of the system were constructed using PACKMOL^68^ to randomize the starting positions of the lipids.

Each system was energy minimized using the steepest descent algorithm, followed by a 100 ps *NVT* equilibration run and a 2 ns semi-isotropic *NPT* equilibration run. Production run simulations were performed in the *NPT* ensemble for 500 ns, with a time step of 2 fs. The temperature of the lipids and water was controlled separately by the Nosé–Hoover thermostat at 305 K, with a time constant of 1 ps. The pressure was controlled semi-isotropically using the Parrinello–Rahman barostat, with a time constant of 5 ps and compressibility of 4.5 × 10^–5^ bar^–1^. All lipid bonds were constrained using the LINCS algorithm. Electrostatic and van der Waals interactions were calculated using the PME method with a short-range cutoff distance of 1.2 nm and a force switch from 1 to 1.2 nm for van der Waals interactions. PBCs were applied in the *x*, *y*, and *z* directions.

### 2.5 Bilayers Solvated with PG

The equilibrated CHARMM and UA bilayers were solvated with PG and water using the GROMACS “insert_molecules” module to give PG concentrations of 20, 40, 60, 80, and 100% w/w. The overall ratio of lipid:solvent remained 1:30. Table 1 shows the PG and water composition of the solvated bilayers simulated in this work. Two additional repeat simulations were performed for each of the CHARMM and UA systems containing 20 and 80% PG by solvating the replica bilayers generated previously.

**Table 1:** Number of PG and Water Molecules Used to Solvate the CHARMM and UA Bilayers with 0–100% w/w PG

system	PG concentration (% w/w)	no. PG molecules	no. water molecules
CHARMM	0	0	8640
	20	438	8157
	40	1179	7461
	60	2265	6375
	80	4204	4436
	100	8640	0
UA	0	0	5400
	20	326	5074
	40	790	4610
	60	1503	3897
	80	2739	2661
	100	5400	0

Each system was energy minimized using the steepest descent algorithm, followed by a 1 ns *NVT* equilibration run and a 10 ns semi-isotropic *NPT* equilibration run. Production run simulations were performed in the *NPT* ensemble for 500 ns at 305 K and 1 bar pressure. A time step of 2 fs was used. The temperature of the lipids and solvent (water and PG) was controlled separately using the Nosé–Hoover thermostat with a time constant of 0.5 ps. The pressure was controlled semi-isotropically using the Parrinello–Rahman barostat, with a time constant of 2 ps and compressibility of 4.5 × 10^–5^ bar^–1^. Bonds involving hydrogen were constrained using the LINCS algorithm. Electrostatic and van der Waals interactions were calculated using the PME method with a short-range cutoff distance of 1.2 nm and a force switch from 1 to 1.2 nm for van der Waals interactions. PBCs were applied in the *x*, *y*, and *z* directions.

Additional longer simulations were performed whereby the 500 ns production run of the CHARMM and UA systems containing 80% PG were extended to give a total production run of 2 μs. All other simulation parameters used were the same as those used for the initial 500 ns simulations. A system containing 46 PG molecules starting inside the bilayer was also simulated to determine whether the initial position of PG impacts its partitioning behavior (see Section S7).

To ensure that our results were not highly dependent on the choice of ceramide species, an additional AA model bilayer composed of CER[NP]24 (Figure S21), FA24, and CHOL in a 1:1:1 ratio was solvated with 80% w/w PG and simulated for a production run of 500 ns, using the same simulation parameters as the corresponding CER[NS]24 system. Note that CER[NP]24 refers to a phytosphingosine (P) base connected to a non-hydroxy fatty acid (N) by an amide bond.

### 2.6 Analysis

All analyses were performed on the last 400 ns of the production run simulations, and averages and error bars were obtained by block averaging over ten 40 ns blocks. Analysis of the area per lipid (APL) over time to determine whether each system had equilibrated in the 500 ns simulation times is detailed in Section S2, and analysis of the repeat simulations can be found in Section S4.2. The APL was calculated by dividing the area of the simulation box in the *xy*-plane by the total number of lipids per leaflet. Density profiles were calculated using the GROMACS “density” module by splitting the trajectory of each system into 50 slices along the *z*-coordinate and then calculating the average density of each species within each slice. The order parameter *S*_*z*_ for atom C*_n_* was calculated for the CER[NS]24 sphingosine and fatty acid chains and the FA24 chain according to
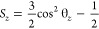
3where θ_*z*_ is the angle between the *z*-axis (bilayer normal) and the vectors C*_n_*_–1_ to C*_n_*_+1_. *S*_*z*_ can take values between 1 and −0.5, which correspond to the lipid chain being parallel to and perpendicular to the bilayer normal, respectively. H-bonds were calculated using the GROMACS “hbond” module, whereby H-bonds are defined to exist if the distance between donor and acceptor atoms is less than 0.35 nm, and if the hydrogen-donor···acceptor angle is less than 30°. The bilayer thickness was calculated from the distance between the two peaks of the CER[NS]24 headgroup density profiles. The potential of mean force (PMF) of water as a function of the distance from the bilayer center was calculated from the water density (ρ_w_) profiles using the equation below
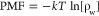
4where *k* is the Boltzmann constant, and *T* is the system temperature. The one-way analysis of variance (ANOVA) test was employed to evaluate the statistical significance of calculated properties, with posthoc pairwise comparisons conducted using Tukey’s honestly significant difference (HSD) test. The analysis was performed using the scipy.stats SciPy version 1.1.4 Python package, with the significance level set to 0.05.

## 3 Results

### 3.1 PG Model Validation

**Figure 3:**
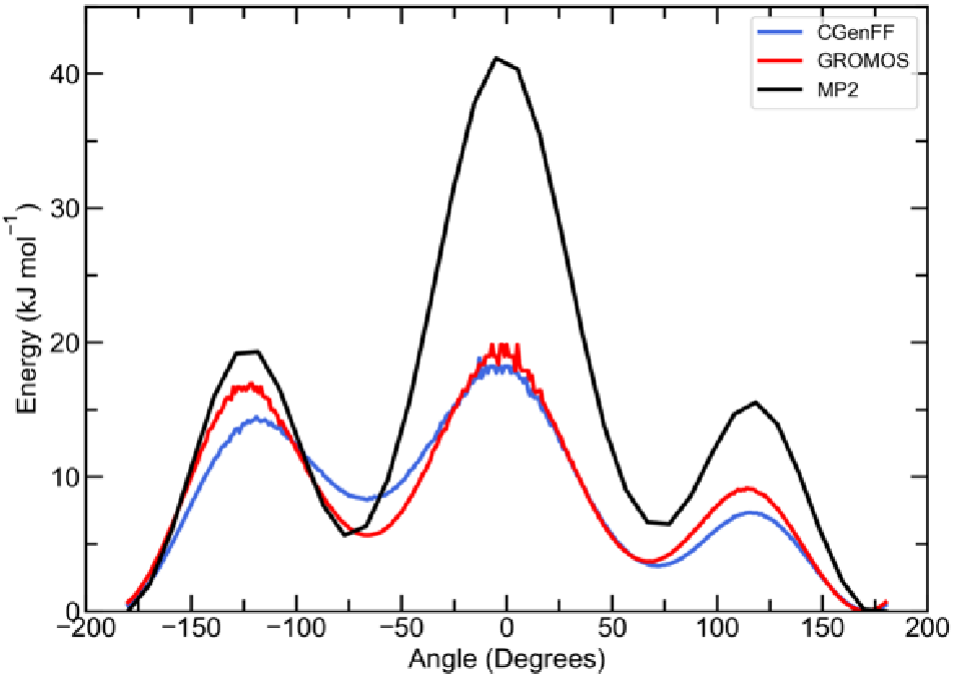
Energy profile of the O–C–C–O dihedral angle of PG obtained from MP2 calculations (black) and of the CGenFF (blue) and GROMOS (red) models of PG obtained from MD simulations of pure PG.

Various properties of PG were evaluated to validate the CGenFF and GROMOS models of PG against experimental data. Table 2 shows the density of the two models of PG as well as the corresponding experimental value. The simulated densities of PG at 298.15 K using both force field models are in good agreement with experimental data, with the CGenFF model having the closest agreement. Table 2 also shows the computed log *P* values of the CGenFF and GROMOS PG models, as well as the experimental value. Both force fields have a less negative log *P* than expected, indicating that the PG models used in this work are more hydrophobic than real PG. However, this does not appear to influence the partitioning behavior of PG into the skin lipid membranes to the extent that PG preferentially partitions into the bilayer, as discussed in Section S3. The structure of both PG models was also analyzed to confirm that the O–C–C–O dihedral angle behaves in accordance with the energy profile obtained from quantum mechanical calculations (Figure 3). For both PG force field models, the energy profiles obtained from the MD simulations are a similar shape to that obtained from quantum mechanical calculations, with peaks centered around 0° and ±125°, confirming that both the CGenFF and GROMOS PG models reproduce the O–C–C–O dihedral angle behavior expected from quantum mechanical calculations. Further validation of the structure and properties of the PG models, including the self-diffusion coefficient, radial distribution functions (RDFs), and H-bonds can be found in Section S1.2–1.4.

**Table 2:** Simulated Density and Log *P* Values of the CGenFF and GROMOS Models of PG, as Well as the Corresponding Experimental Value

	property	
system	density/kg m^–3^	log *P*
CGenFF	1018.88	–0.70
GROMOS	1090.78	–0.53
experiment	1033.00^69,70^	–0.92^71−73^

### 3.2 Properties of the Bilayers Solvated with 0–100% PG

#### 3.2.1 Area Per Lipid

The APL gives insights into the packing of the lipid headgroups. The APL of the CHARMM bilayer solvated with pure water was calculated to be 0.325 ± 0.001 nm^2^, which is in good agreement with that obtained by Piasentin et al.^54^ (0.325 ± 0.002 nm^2^), who also used the CHARMM36 force field to study an equimolar CER[NS]24/FA24/CHOL bilayer at 303.15 K, and the APL value of 0.307 ± 0.0003 nm^2^ for the UA bilayer in pure water is in good agreement with that obtained from MD simulations by Del Regno and Notman^55^ (0.304 ± 0.02 nm^2^) using the same model bilayer at 305 K.

**Figure 4:**
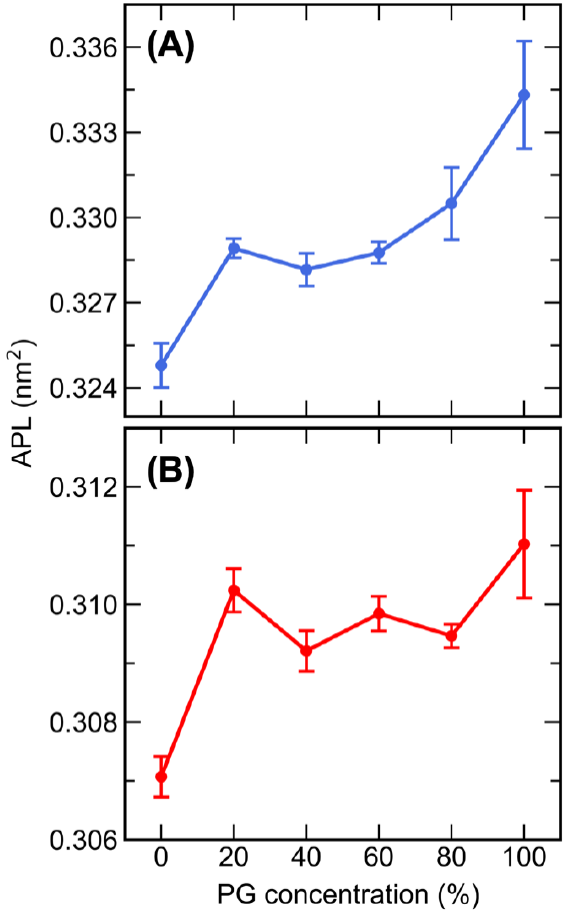
Average APL of the (A) CHARMM and (B) UA bilayers solvated with 0–100% w/w PG.

For both the CHARMM and UA systems, a small but statistically significant (*p* < 0.05) increase in the APL was observed upon the addition of PG (Figure 4). As the concentration of PG was increased from 40 to 100%, there was a slight increase in the APL in the CHARMM bilayer, whereas no clear trend was observed for the APL of the UA bilayer as the concentration of PG was increased. Both systems showed an additional increase in the APL for the pure PG systems compared to pure water or PG/water mixtures. A similar effect of PG on the APL of phospholipid bilayers in the liquid crystalline phase has also been observed, whereby increasing the concentration of PG caused an increase in the APL of pure DOPC^37^ and DPPC^38^ bilayers. The effect on the APL is likely due to the interaction of PG with the lipid headgroups, where it disrupts headgroup–headgroup and headgroup–water interactions, as discussed in Section 3.2.5.

#### 3.2.2 Bilayer Thickness

The bilayer thickness provides insights into the organization of lipid tails. A decrease in bilayer thickness may correspond to increased interdigitation or disordering of lipid tails. For the pure water systems, the CHARMM bilayer thickness value of 4.89 ± 0.04 nm is in good agreement with that of Piasentin et al.^54^ (4.90 ± 0.04 nm), and the UA bilayer thickness value of 4.71 ± 0.08 nm is in good agreement with that obtained by Del Regno and Notman^55^ (4.76 ± 0.3 nm). The bilayer thickness values for both pure water systems are close to the experimental value of 5.39 nm observed by Školová et al.^74^ when studying an equimolar CER[NS]/FA24/CHOL membrane at 330.15 K.

**Figure 5:**
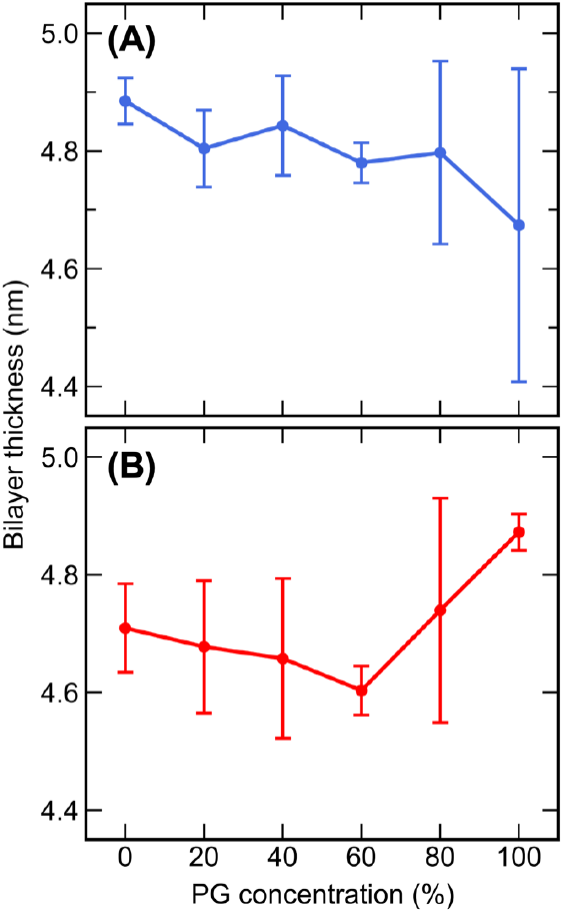
Average bilayer thickness of the (A) CHARMM and (B) UA bilayers solvated with 0–100% w/w PG.

The bilayer thickness of the CHARMM and UA systems for each PG concentration studied is shown in Figure 5. In the CHARMM systems, the bilayer thickness was found to decrease slightly for all concentrations of PG; however, computation of errors from block averaging and statistical tests showed that these results are not statistically significant (*p* > 0.05), except for the difference between 0 and 100% PG (*p* < 0.05). In the UA systems, the bilayer thickness decreased slightly for concentrations of 20–60% PG and increased slightly for 80 and 100% PG. However, similarly to the CHARMM systems, these results do not appear to be statistically significant (*p* > 0.05), except for the increase in bilayer thickness between 0 and 60% PG with 100% PG (*p* < 0.05). Overall, for both force fields, the addition of PG did not appear to have a significant impact on the bilayer thickness.

#### 3.2.3 Density Profiles

**Figure 6:**
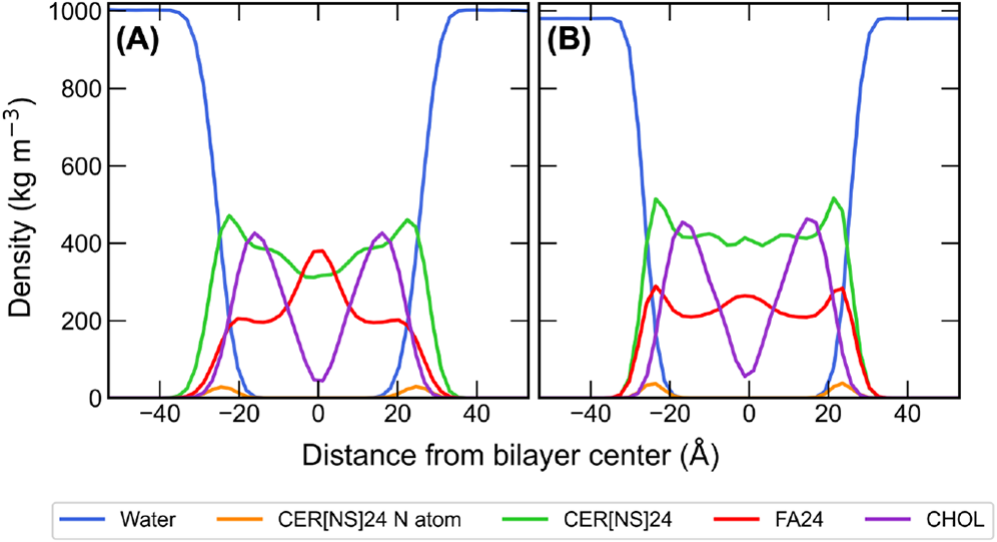
Density profiles of water (blue), CER[NS]24 (green), FA24 (red), CHOL (purple), and CER[NS]24 N atoms (orange) in the (A) CHARMM and (B) UA systems solvated with pure water.

The density profiles in Figure 6 show the molecular density of each component of the CHARMM and UA bilayers solvated with pure water as a function of their position along the bilayer normal (*z*-axis). Overall, the shape of the density profiles of each component is similar for the CHARMM and UA bilayers. The FA24 density profiles show regions of higher density at the center of the bilayer due to tail interdigitation, which is more pronounced in the CHARMM bilayer. CHOL molecules tend to sit slightly below the interface, oriented so that their hydroxyl group is aligned with the CER[NS]24/FA24 headgroup region, and due to their shorter length, they have a much lower density than the CER[NS]24 and FA24 molecules at the center of the bilayer.

**Figure 7:**
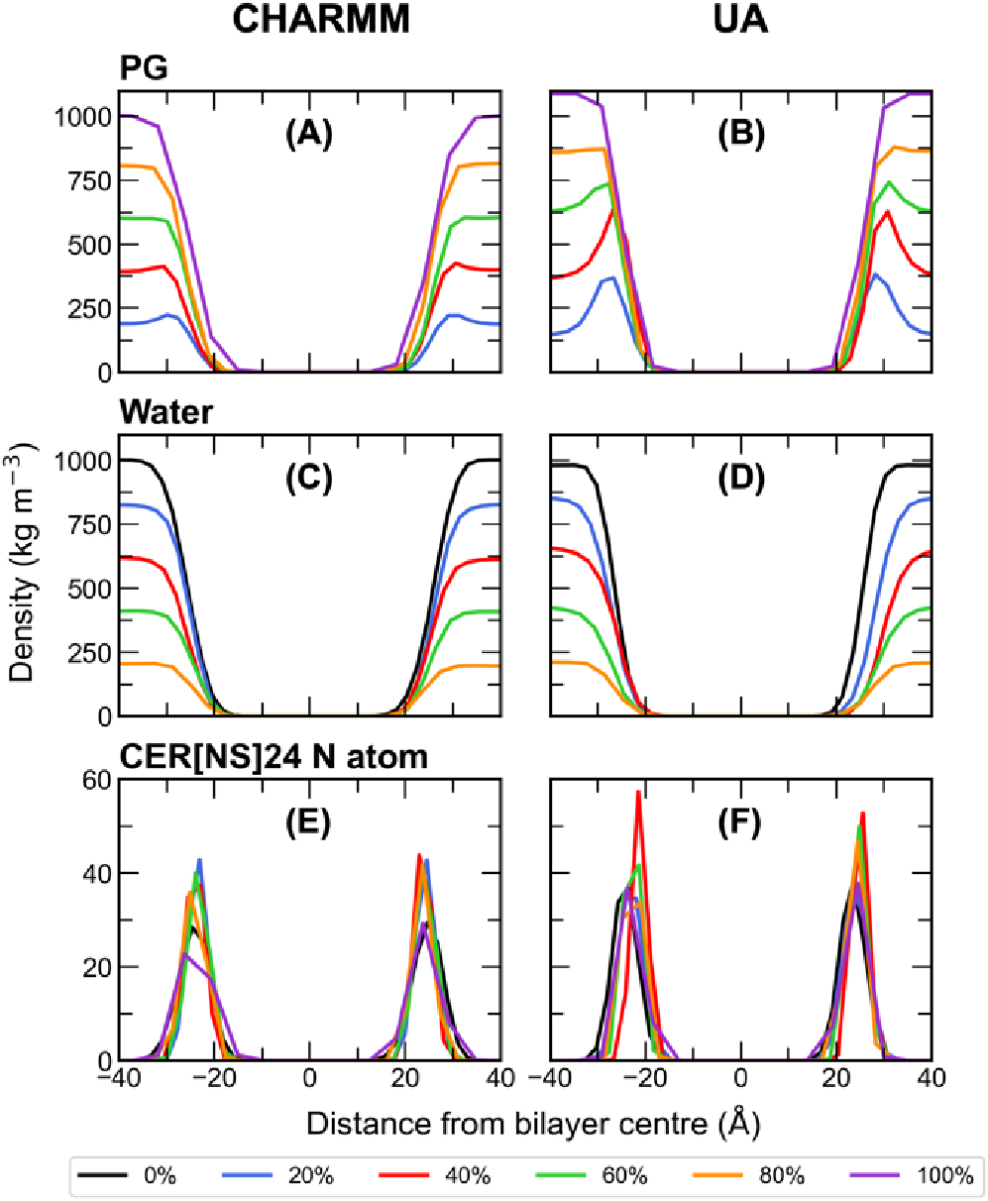
Density profiles of (A) PG, (C) water, and (E) CER[NS]24 N atoms in the CHARMM bilayers solvated with 0–100% w/w PG and of (B) PG, (D) water, and (F) CER[NS]24 N atoms in the UA systems solvated with 0–100% w/w PG.

When PG is introduced into the CHARMM and UA systems, we observe that PG remains in the aqueous phase rather than spontaneously partitioning into or permeating across the bilayer (see Figures S6 and S7 for selected snapshots of each system). Visual inspection of the trajectories shows that at low concentrations of PG, there is an accumulation of PG at the bilayer interface (headgroup region), which is quantitatively confirmed by the system density profiles (Figure 7). At concentrations up to 60% w/w PG in the UA system and 40% w/w in the CHARMM system, there are peaks in the density profiles centered on the headgroup regions, confirming that PG localizes at the bilayer interface. The density is zero between the headgroup peaks, which confirms that PG did not sample the bilayer interior on the time scale of the simulation. Therefore, we expect that PG permeation is a relatively rare event. At higher concentrations of PG, there is a smooth transition across the bilayer interface from the bulk PG concentration to zero beneath the lipid headgroups. At each concentration of PG, the UA density profiles for PG have a higher density at the interface than the equivalent CHARMM system. This suggests that PG in the UA systems has a slightly greater affinity for the interface than it does in the CHARMM systems, which seems to be due to a greater proportion of lipid–water H-bonds being replaced with lipid–PG H-bonds in the UA model compared to the AA model.

**Figure 8:**
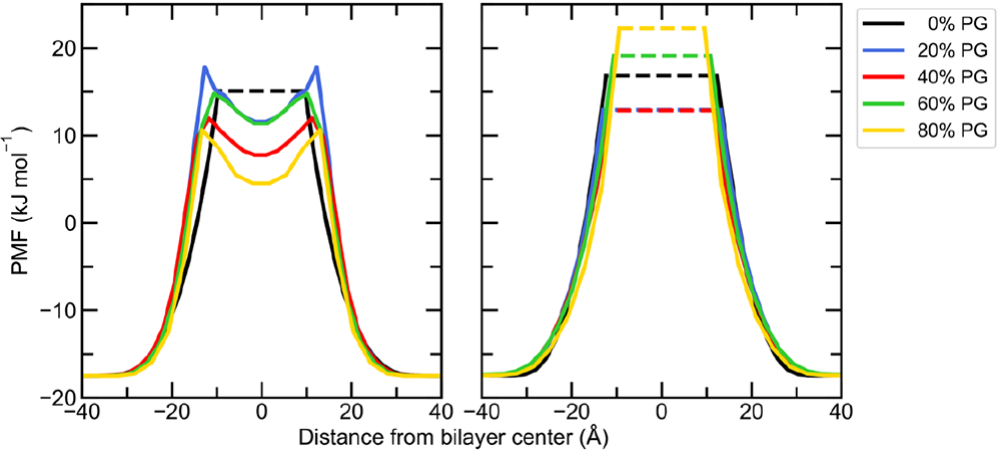
PMF of water as a function of the distance from the bilayer center for the (A) CHARMM and (B) UA systems solvated with 0–100% PG. Dashed lines are shown in the regions where the density of water was zero.

Figure 8 shows the PMF of water as a function of the distance from the bilayer center for each system. In the absence of PG, no water is observed in the central region of the CHARMM and UA bilayers. However, some water is found to enter the bilayer in the presence of PG in the CHARMM systems, and the PMF barrier height decreases with increasing PG concentration, which is direct evidence that PG enhances the permeation of water into the bilayer. For the UA systems, no water is observed inside the bilayer for any concentration of PG. This implies that there is a higher barrier to water permeation in the UA force field, which would require enhanced sampling techniques to quantify.

#### 3.2.4 Order Parameters

**Figure 9:**
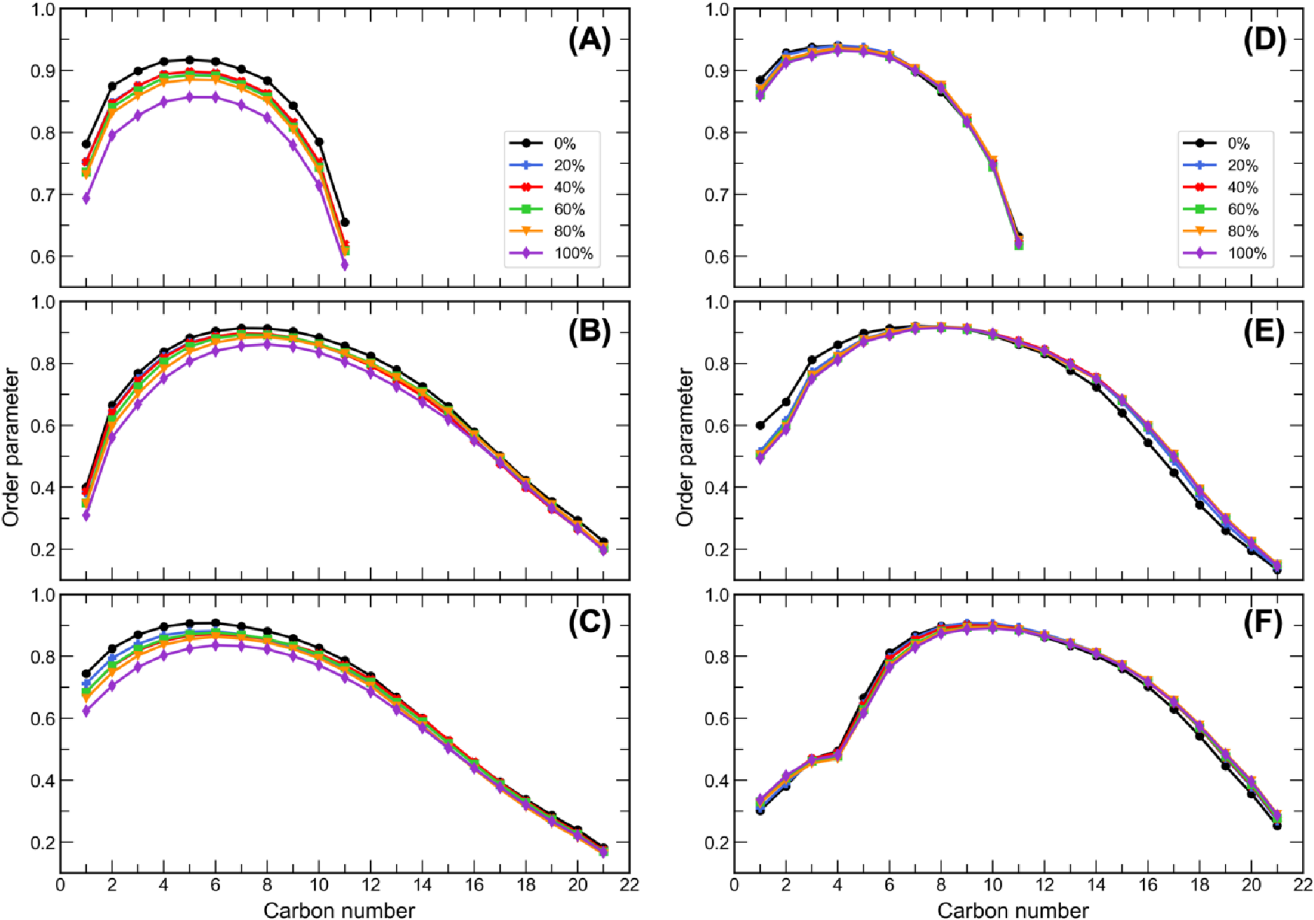
Lipid-tail order parameters for the (A) sphingosine and (B) fatty acid chains of CER[NS]24 and (C) FA24 in the CHARMM bilayers solvated with 0–100% w/w PG and for the (D) sphingosine and (E) fatty acid chains of CER[NS]24 and (F) FA24 in the UA bilayers solvated with 0–100% w/w PG. Error bars are not shown on the graphs but were all calculated to be ≤0.01.

**Figure 10:**
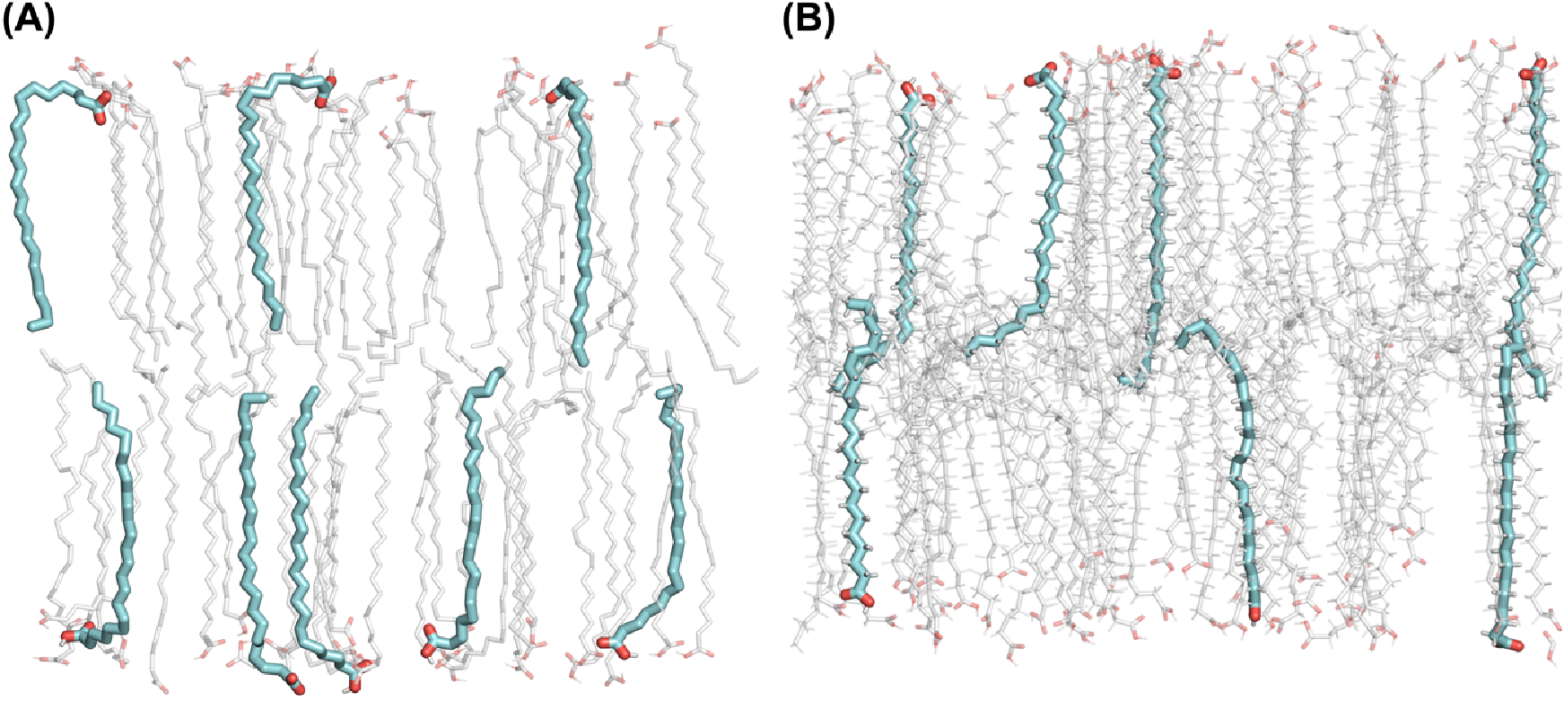
Snapshots of FA24 molecules in the (A) UA and (B) CHARMM bilayers solvated with pure water, highlighting the tendency for UA FA24 molecules to bend at the interface, whereas CHARMM FA24 molecules prefer to align themselves with the *z*-axis.

Lipid tail order parameters provide a measure of the orientation and alignment of lipid tails with respect to the bilayer normal. The order parameters of the CER[NS]24 sphingosine and fatty acid chain C atoms and the FA24 chain C atoms are shown in Figure 9 for the CHARMM and UA bilayers solvated with 0–100% PG. In the systems solvated with pure water, the CER[NS]24 sphingosine and fatty acid chain order parameters are similar for the CHARMM and UA force fields. Both chains show moderate disorder in the atoms closest to the interface. The tails then become more ordered moving down the chain, peaking at carbon 9, and become disordered again in the region at the center of the bilayer. The same behavior is observed for the FA24 chain; however there appears to be a higher degree of disorder near the interface for the UA FA24 chain than the CHARMM FA24 chain. This may be explained by the way that the system minimizes the hydrophobic mismatch between the length of the FA24 chain and the length of the dense region of the bilayer. In the UA model, we frequently observe bending of the FA24 molecules at the interface (see Figure 10A). This is observed less frequently in the CHARMM membrane (see Figure 10B), where the FA24 molecules consistently penetrate deeper into the bilayer with a greater degree of interdigitation in the center of the bilayer. This is reflected by the higher peak in the density of the FA24 in the center of the bilayer in the CHARMM membrane; hence, the atoms closer to the interface are more ordered.

In the CHARMM systems, the order parameters of all three lipid tails decreased with increasing PG concentration. For the CER[NS]24 sphingosine tails, increasing the PG concentration caused a decrease in the order parameter of each C atom to a similar extent. However, for the CER[NS]24 fatty acid tail and the FA24 tail, the order parameters of C atoms further up the chain (closer to the interface) were decreased by a greater amount than those deeper inside the bilayer. This suggests that PG had a greater disordering effect on the atoms closer to the interface. PG was also found to affect the order parameters of UA lipid tails in a concentration-dependent manner, although to a lesser extent. For the CER[NS]24 sphingosine tail, the order parameters decreased slightly with increasing PG concentration. Interestingly, for the CER[NS]24 fatty acid tail and the FA24 tail, increasing the concentration of PG caused the order parameters to decrease for C atoms further up the chains (<C9) and increase for C atoms deeper inside the bilayer (>C13). This suggests that the lipid tails that lie deeper inside the bilayer become slightly more ordered in the presence of PG. Overall, PG caused a slight disruption to the lipid tail organization in the CHARMM and UA systems, with a greater disordering effect being observed for the CHARMM systems. It should be noted that the lipids remained in an ordered gel phase, despite PG slightly disrupting their organization.

#### 3.2.5 Hydrogen Bonding

**Figure 11:**
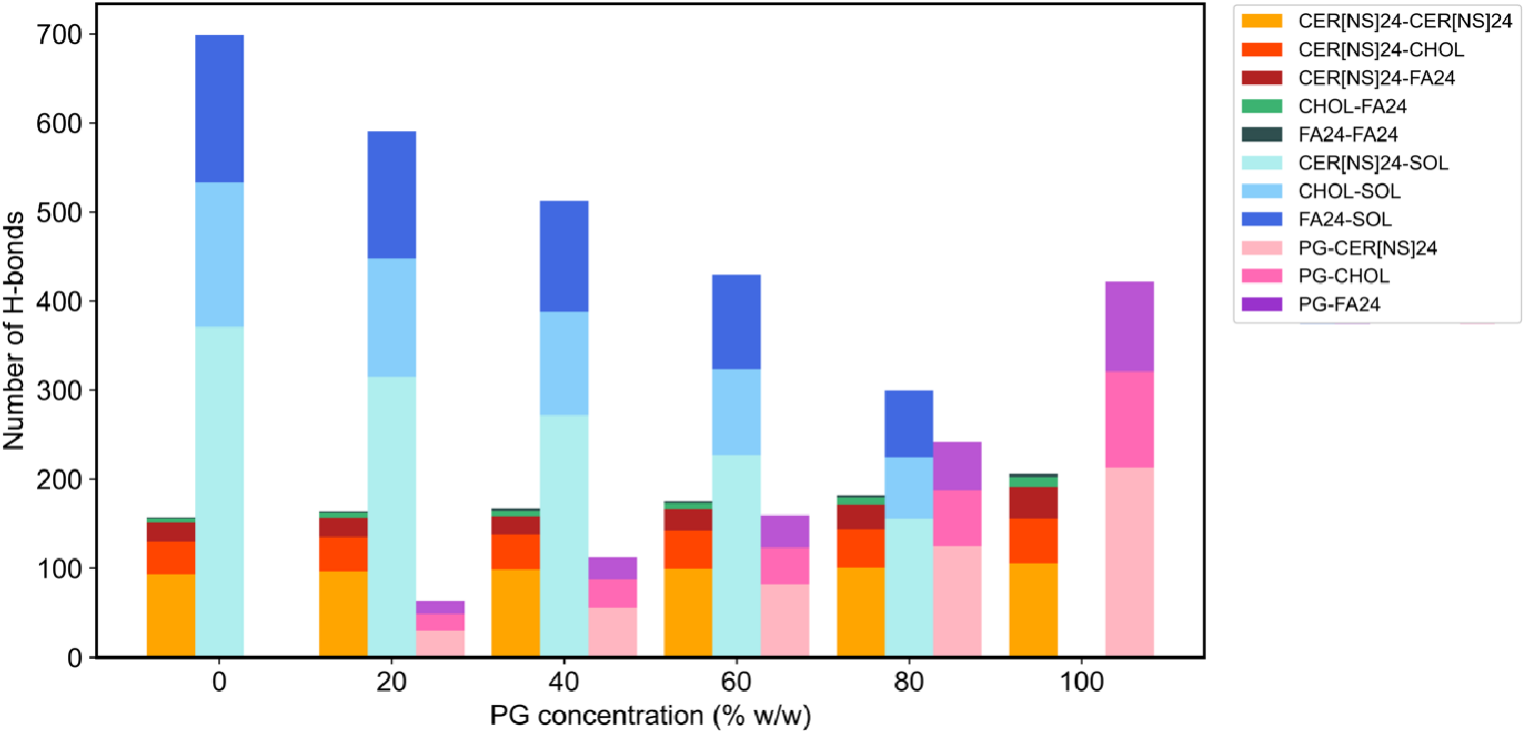
Number of lipid–lipid and lipid–solvent H-bonds present in the CHARMM systems solvated with 0–100% w/w PG.

**Figure 12:**
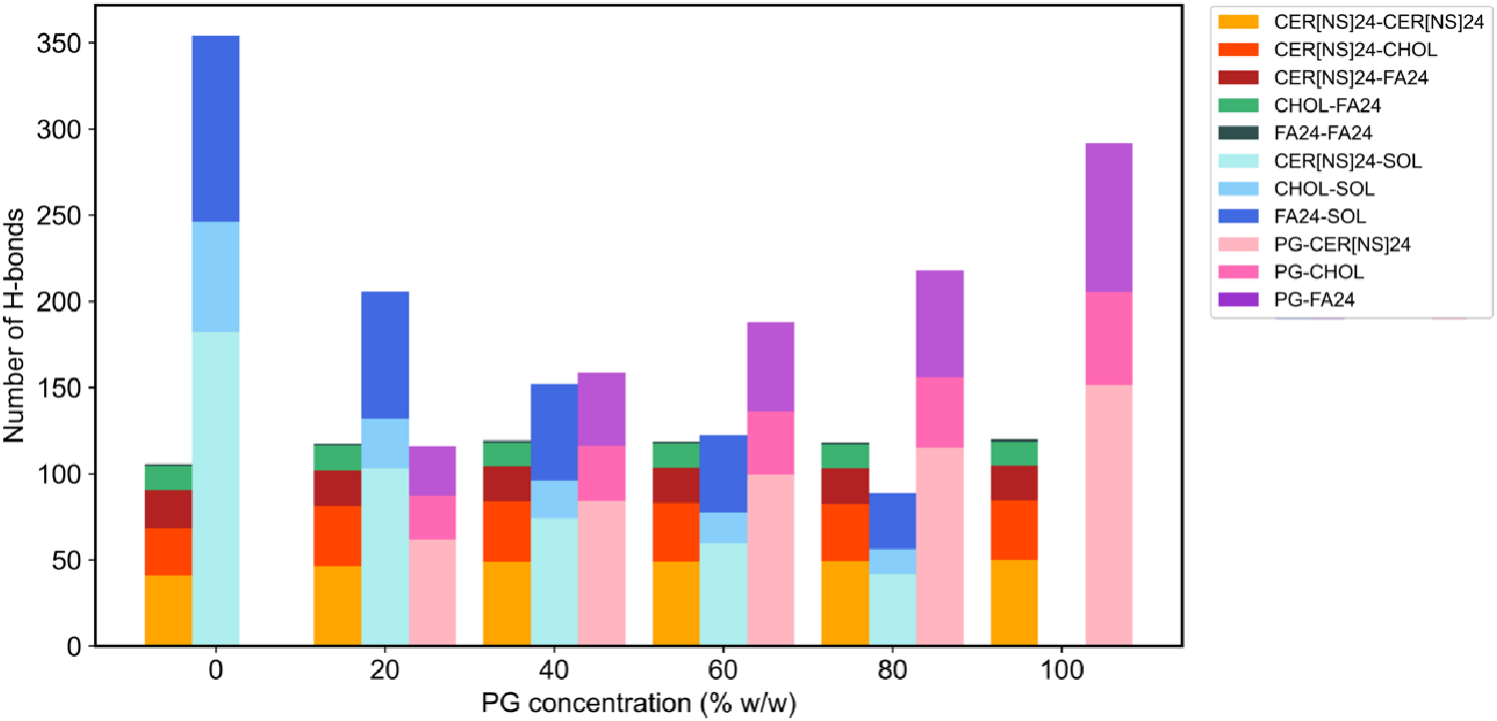
Number of lipid–lipid and lipid–solvent H-bonds present in the UA systems solvated with 0–100% w/w PG.

The strong barrier properties of the SC lipids may be attributed to the lateral H-bond network between the lipid headgroups.^64^ A possible penetration-enhancing mechanism of PG is to disrupt this H-bond network; therefore, the H-bond network of the lipids in the presence of PG was investigated. The number of H-bonds between each species in the CHARMM and UA systems is shown in Figures 11 and 12. In both the CHARMM and UA systems, PG was able to form H-bonds with each class of lipid, and as the concentration of PG increased, the number of PG-lipid H-bonds increased. At every PG concentration studied, PG formed more H-bonds to CER[NS]24 than FA24 or CHOL, which is likely due to the greater number of H-bond donor/acceptor atoms in CER[NS]24 than the other lipids. The number of lipid–water H-bonds decreased with increasing PG concentration, and at each concentration of PG, PG molecules formed more H-bonds to water molecules than to other PG molecules (see Figure S10). In the CHARMM systems, the total number of lipid–lipid H-bonds increased slightly with increasing PG concentration, whereas in the UA systems, the total number of lipid–lipid H-bonds was slightly increased to a similar extent across all concentrations of PG. All in all, the effect of PG is to disrupt bilayer–water H-bonds while having a minimal effect on the lipid–lipid H-bonding network.

## 4 Discussion

### 4.1 PG Partitioning Behavior

PG is a widely used skin penetration enhancer that is utilized in TDD systems to overcome the skin’s main permeability barrier, the SC. Although the use of PG as a penetration enhancer is widely documented in the literature, little is known about the penetration-enhancing mechanism of PG at the molecular level. However, experimental studies suggest that it is likely to involve interaction with the SC lipids. Therefore, the purpose of this study was to investigate the effects of PG on the structure and properties of model skin lipid bilayers at the molecular level. First, we constructed AA and UA models of an SC bilayer and simulated them in water, and then, we investigated the interactions of PG with the equilibrated model bilayers by solvating them with concentrations of PG from 20 to 100% w/w and performing further MD simulations.

We observe that, on the time scales of our simulations, PG preferentially accumulates at the bilayer interface, rather than partitioning into the bilayer. This behavior is observed at every concentration. The tendency for PG to localize at the interface in these simulations may be rationalized by its polarity. PG is a moderately polar molecule, with octanol/water log *P* values of −0.70 and −0.53 for the CHARMM and UA models, respectively; therefore, it is likely to have a higher affinity for the hydrophilic bilayer interface than the hydrophobic bilayer interior. We also find that PG increases the APL of the bilayers, which is likely due to PG accumulating in the headgroup regions and forming H-bonds with the lipids, resulting in a slight lateral expansion of the interface to accommodate the PG molecules. The partitioning behavior of PG observed in our work is similar to that seen of PG interacting with phospholipid bilayers. In MD simulations performed by Malajczuk et al.^38^ and Hughes et al.,^37^ PG was found to aggregate in the lipid headgroup regions of DPPC and DOPC bilayers, causing an increase in the APL.

The observation that PG localizes in the lipid headgroup regions has been demonstrated previously in experiments.^7,33^ Brinkmann et al.^33^ used SAXD to analyze human SC treated with PG and found that PG was able to insert into the headgroup regions. Similarly, Bouwstra et al.^7^ used SAXS and differential thermal analysis (DTA) to study the effects of PG and water on the properties of human SC. They suggested that PG is able to intercalate into the lipid headgroup regions of the SC, leading to an increase in the interfacial APL.

Experimental studies have also shown that PG is able to penetrate skin and partition into the SC. For example, in a series of experiments performed by Nicoli et al.^75^ in which ibuprofen in PG/water mixtures was applied to human skin, ATR-FTIR spectroscopy was able to detect PG in the SC and monitor PG profiles across the SC using tape-stripping. Similarly, Pudney et al.^30^ used *in vivo* confocal Raman spectroscopy to reveal that PG penetrates human skin into the SC. The rate of PG penetration was found to increase with time, with PG being detected in the SC at depths greater than 8 μm for 6 h after initial application. We do not observe PG permeation in our simulations, but it is very likely that PG will permeate the bilayer as a rare event with a multiple kJ mol^–1^ barrier, which would require enhanced sampling to approximate. Based on the results of our simulations and previous experimental studies in the literature, it is likely that PG permeates through the SC lipids and associates near the lipid headgroups.

Although experiments such as those described above show that PG diffuses through skin, it should be noted that the exact transport pathway of PG remains unknown, though it is likely to pass through the lipids since they provide the only continuous pathway through the SC. Since real skin lipid bilayers are highly heterogeneous, the transport pathway of PG may actually be through defects or nanoscale domains enriched with certain lipid species, which are not captured by the “idealized” model skin lipid bilayers that are often used in simulations. Additionally, our model skin lipid bilayers greatly oversimplify the structure and composition of the SC lipid layers since only three different lipids are used. Therefore, models that are better able to capture the complexity of the SC, such as those composed of several classes of CERs, FFAs, and CHOL, or with the presence of microscopic defects, may be required to observe PG permeation. With this in mind, we built and solvated an additional AA model bilayer composed of CER[NP]24/FA24/CHOL (1:1:1) with 80% w/w PG to see if any differing PG partitioning behavior would be observed. This bilayer composition may be a more representative model of the SC since CER[NP]24 has been found to be more abundant in human SC than CER[NS]24.^76,77^ However, PG displayed the same partitioning behavior in this system as that of the equivalent CER[NS]24/FA24/CHOL system, with a preference to localize in the lipid headgroup regions rather than permeating the bilayer (see Section S5). The lipid arrangements in the long periodicity phase and the presence of ceramides in an extended conformation (rather than in a hairpin conformation as simulated in this work) might also affect how PG interacts with more complex SC bilayers. We modeled our bilayers with excess water, whereas the hydration level of real SC lipid lamellae is lower (<5 waters per lipid). Therefore, it is possible that PG may have a different effect on low hydration SC bilayers. The fatty acids simulated in this work are uncharged; however, a pH gradient exists across the SC, and there will likely be a fraction of charged FFAs, which may also influence the way PG interacts with SC lipid bilayers.

As mentioned above, permeation of PG is likely to be a rare event on the time scale of our simulations. PG diffusion across DOPC bilayers has been observed by Hughes et al. during 300 ns MD simulations; however, these membranes were in the fluid phase. To determine whether longer simulations would enable us to observe spontaneous penetration, the CHARMM and UA simulations with 80% PG were extended to give a total simulation time of 2 μs. However, PG still did not permeate the bilayer in these extended simulations (see Figures S22 and S23). Interestingly, in the simulation with PG starting inside the bilayer, all PG molecules were able to exit the bilayer within 500 ns, where they then remained in the solvent phase. Therefore, it is likely that the relatively large free energy barrier to permeation is associated with PG moving from the aqueous phase, across the headgroups, into the dense lipidic phase, which results in a low probability of spontaneous permeation of PG on the time scales simulated in this work.

### 4.2 Effect of PG on the Structure and Properties of the Membrane

While PG was able to form H-bonds with the CER[NS]24, FA24, and CHOL headgroups, overall, the H-bond network between the lipids remained intact. In fact, the overall number of H-bonds between the lipids was found to increase in the presence of PG. Instead, PG was found to disrupt lipid–water H-bonds, with higher concentrations of PG causing a greater reduction in the number of lipid–water H-bonds. This is likely due to PG occupying the lipid–water H-bond sites instead. Similar behavior has also been observed in MD simulation studies of PG interacting with DOPC and DPPC lipid bilayers. Hughes et al.^37^ reported a decrease in the number of DOPC–water H-bonds due to the accumulation of PG at the bilayer interface, while Malajczuk et al.^38^ found that the number of DPPC–water H-bonds decreased with increasing PG concentration.

From the order parameters of the CER[NS]24 and FA24 tails, we find that PG has a slight disordering effect on the lipid bilayers. The greatest disordering effect is achieved near the interface in the upper portion of the lipid tails, which is expected since PG localizes in the lipid headgroup regions. As PG does not penetrate the bilayer, it has less of a disordering effect on the parts of the lipid tails that lie deeper inside the bilayer. Carrer et al.^31^ used μFTIR spectroscopy to analyze pig skin treated with PG and concluded that PG was able to alter the barrier function of skin by affecting the bilayer structure of intercellular lipids and by increasing lipid disorder in the epidermis. Our results are consistent with these experimental observations.

## 5 Conclusion

In this study, we have carried out MD simulations of model SC bilayers with different concentrations of PG (0–100% w/w), using two different force fields. Rather than partitioning into the bilayer, PG tends to accumulate in the headgroup regions at the interface, causing a slight increase in the APL. Here, it is able to occupy lipid–water H-bond sites, but overall causes little change to the H-bond network between the lipids. PG also disrupts the organization of the bilayer by inducing a slight disorder in the lipid tails, with a greater disordering effect observed nearer the interface. This study provides the first insights from molecular simulation into how PG affects the structure and properties of model SC bilayers and how it interacts with SC lipids at the molecular level. The results suggest that interfacial adsorption is an important part of the penetration-enhancing mechanism of PG, which will help to guide the rational design of transdermal formulations that target different aspects of the skin barrier. The work also lays the foundation for future simulation studies of drug permeation in the presence of PG.

## Supporting Information Available

Further validation of the PG models and results from the analysis of repeat simulations, the system where PG started inside the membrane, and the 2 μs simulations of the 80% PG systems (PDF)
Gromacs-compatible topology (.itp) files of AA model for PG (PDF)
Gromacs-compatible topology (.itp) files of UA model for PG (PDF)

## Abbreviations

AA: all-atom
APL: area per lipid
CER[NS]24: ceramide 2
CHOL: cholesterol
DOPC: 1,2-dioleoyl-*sn*-glycero-3-phosphocholine
DPPC: 1,2-dipalmitoyl-*sn*-glycero-3-phosphatidylcholine
FA24: lignoceric acid
FFAs: free fatty acids
MD: molecular dynamics
PBC: periodic boundary conditions
PG: propylene glycol
PME: particle mesh Ewald
RDF: radial distribution function
SAXD: small-angle X-ray diffraction
SAXS: small-angle X-ray scattering
SC: stratum corneum
TDD: transdermal drug delivery
UA: united atom
