# Supplementary material for: Mechanisms of the Drug Penetration Enhancer Propylene Glycol Interacting with Skin Lipid Membranes
Source: J Phys Chem B. 2024 Apr 16;128(16):3885–97. doi: 10.1021/acs.jpcb.3c06784 (PMC11056976; doi:10.1021/acs.jpcb.3c06784)
Supplement: Supplementary file 1 — jp3c06784_si_001.pdf [file jp3c06784_si_001.pdf]

Supporting Information for: Mechanisms of the drug penetration enhancer propylene glycol interacting with skin lipid membranes

*Jade Mistry, Rebecca Notman**

Department of Chemistry, University of Warwick, Gibbet Hill Road, Coventry,

CV4 7AL, UK

Supporting Information

S1 Validation of PG models

S1.1 log P convergence analysis

Initial production runs of the windowed alchemical free energy simulations were performed for 15 ns. The free energy of hydration ($\Delta G_{\text{hydration}}$) and solvation ($\Delta G_{\text{solvation}}$) of PG in the water and octanol systems respectively was calculated every 1 ns to assess whether the simulations had converged with respect to time. The simulations were then run for a further 15 ns and the convergence of $\Delta G_{\text{hydration}}$ or $\Delta G_{\text{solvation}}$ with respect to time was evaluated again in the same way. Figure S1 shows the results of this convergence analysis for both systems. For both forcefields, the standard errors of the $\Delta G_{\text{hydration}}$ or $\Delta G_{\text{solvation}}$ values start to overlap after around 20-25 ns, therefore it is considered that the free energy simulations converged with respect to time within the 30 ns production run.

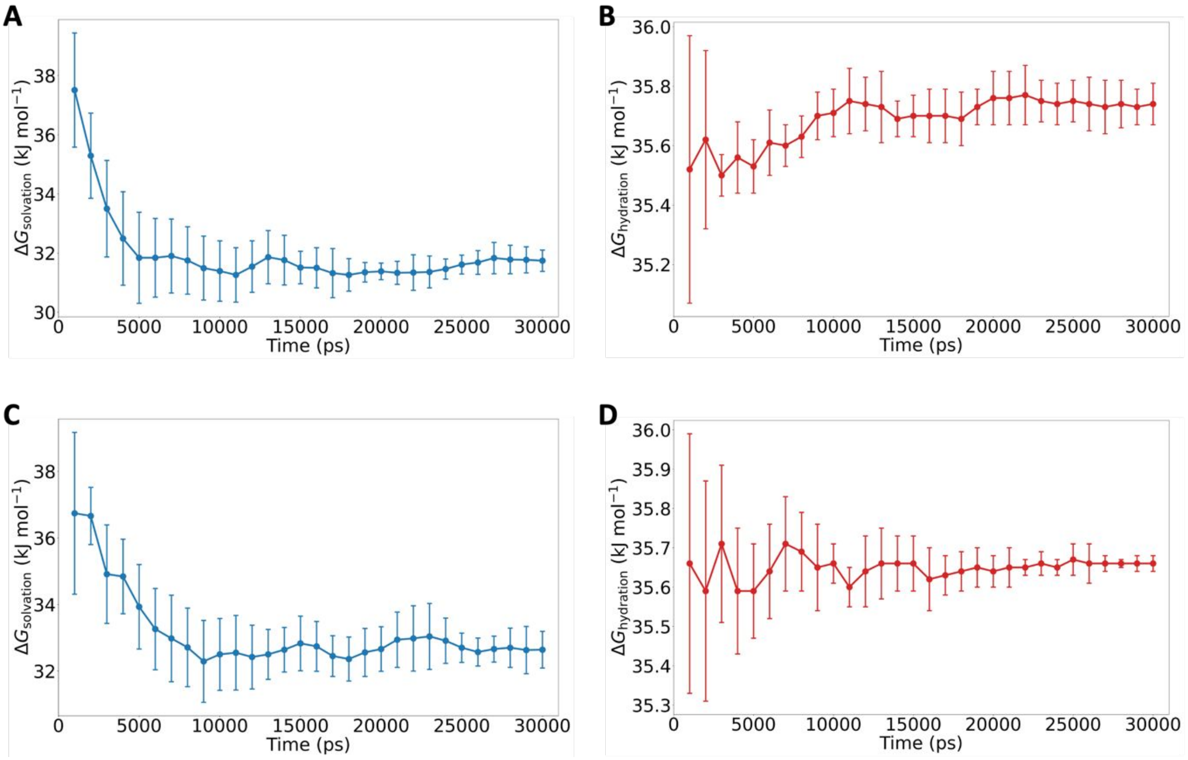

*Figure S1. Convergence of the **(A)** $\Delta G_{solvation}$ and **(B)** $\Delta G_{hydration}$ of PG for the all-atom system and of the **(C)** $\Delta G_{solvation}$ and **(D)** $\Delta G_{hydration}$ of PG for the united atom system.*

S1.2 Self-diffusion coefficient

Table S1 shows the simulated diffusion coefficients of PG at 298.15 K for both forcefield models. Both the CGenFF and GROMOS models slightly overestimated the self-diffusion coefficient of PG compared to the experimental value.

Table S1. Simulated self-diffusion coefficients of the CGenFF and GROMOS PG models, and experimental value.

System	Self-diffusion coefficient /10^{-7} cm^2 s^{-1}
CGenFF	3.33
GROMOS	3.21
Experiment	2.49[1]

S1.3 Radial distribution function

Radial distribution functions (RDFs) for pairs of atoms were calculated using the GROMACS "rdf" module. Figure S2 shows the RDFs for the centre of mass (COM) of the PG molecules at 298.15 K, for both the CGenFF and GROMOS models. The RDFs for the two models of PG are in good agreement with each other, with their peaks and minima at similar positions. The sharp peak at approximately 0.5 nm and the minimum at around 0.7-0.75 nm indicate a well-defined coordination shell. These results are also in good agreement with the RDFs computed by Ferreira *et al.*[2] when parametrising a new forcefield model of PG, indicating both the CGenFF and GROMOS models capture the RDFs and structure of PG well.

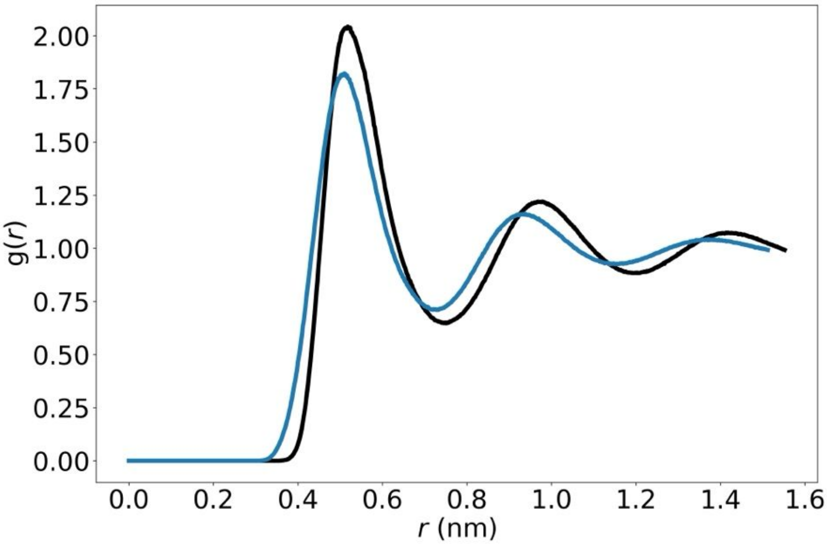

Figure S2. COM RDFs of the CGenFF (black) and GROMOS (blue) models of PG.

S1.4 H-bonds

Table S2 shows the mean relative contribution of H-bonds present in PG. The atom labelling of PG used in this work is shown in Figure S3. A similar H-bond pattern is observed for both forcefields, with the highest contribution to the H-bond interactions coming from the H1-O2 atom pair.

Table S2. H-bonds present in the CGenFF and GROMOS PG models.

System	% H-bonds			
	**O1-H1---O1**	**O1-H1---O2**	**O2-H2---O1**	**O2-H2---O2**
CGenFF	24	27	23	26
GROMOS	24	27	25	25

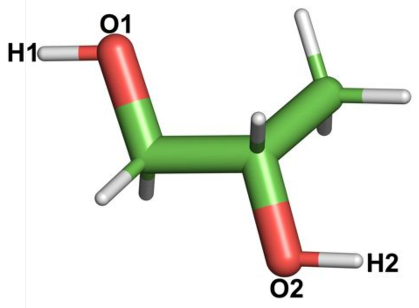

Figure S3. PG atom labelling used in this work.

S2 Equilibration

S2.1 Equilibration of the model bilayers in pure water

The projected area per lipid (APL) of the CHARMM and UA bilayers solvated with water was calculated to determine whether the systems had equilibrated within the 500 ns simulation times. Figure S4 shows that the projected APL for the CHARMM and UA bilayers solvated in water converged within the first 100 ns of the 500 ns production run. This indicates that no significant structural rearrangements were occurring on our simulation timescales, and the systems could be considered equilibrated after 100 ns.

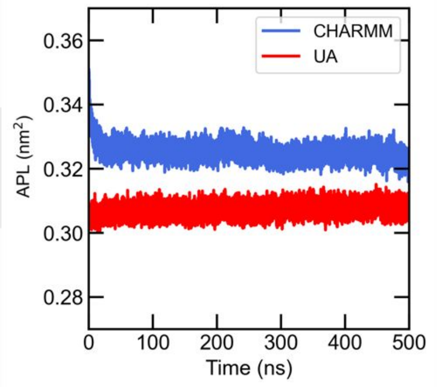

Figure S4. APL over time for the CHARMM (blue) and UA (red) bilayers solvated with pure water.

S2.2 Equilibration of the bilayers solvated with PG

The projected APL of the CHARMM and UA bilayers solvated with 20-100% PG shown in Figure S5 converged within the first 100 ns of the 500 ns production run, indicating that the systems could be considered equilibrated after 100 ns. Note that in simulations of both bilayers in 80% PG, which were extended to 2 µs, the average APL remained the same over this longer timescale.

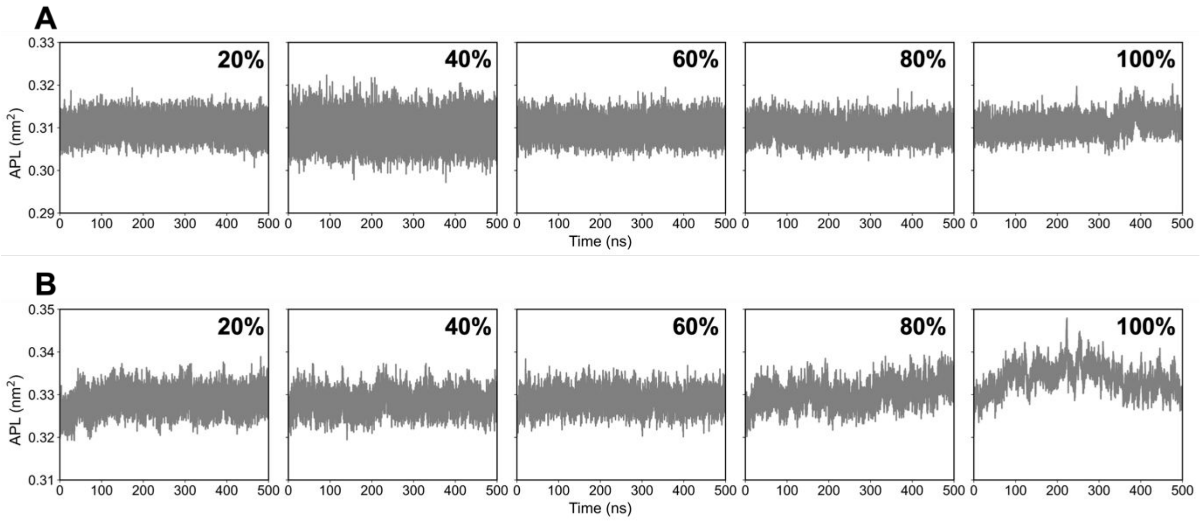

*Figure S5. APL over time of the **(A)** UA and **(B)** CHARMM systems solvated with 20-100% PG.*

S3 PG partitioning behaviour

Snapshots from the trajectories of the CHARMM and UA systems containing 20-100% PG are shown in Figures S6 and S7. We observe that PG remains in the aqueous phase rather than spontaneously partitioning into or permeating across the bilayer. Visual inspection of the trajectories shows that at low concentrations of PG, there is an accumulation of PG at the bilayer interface (headgroup region), which is quantitatively confirmed by the system density profiles (see Section 3.2.3).

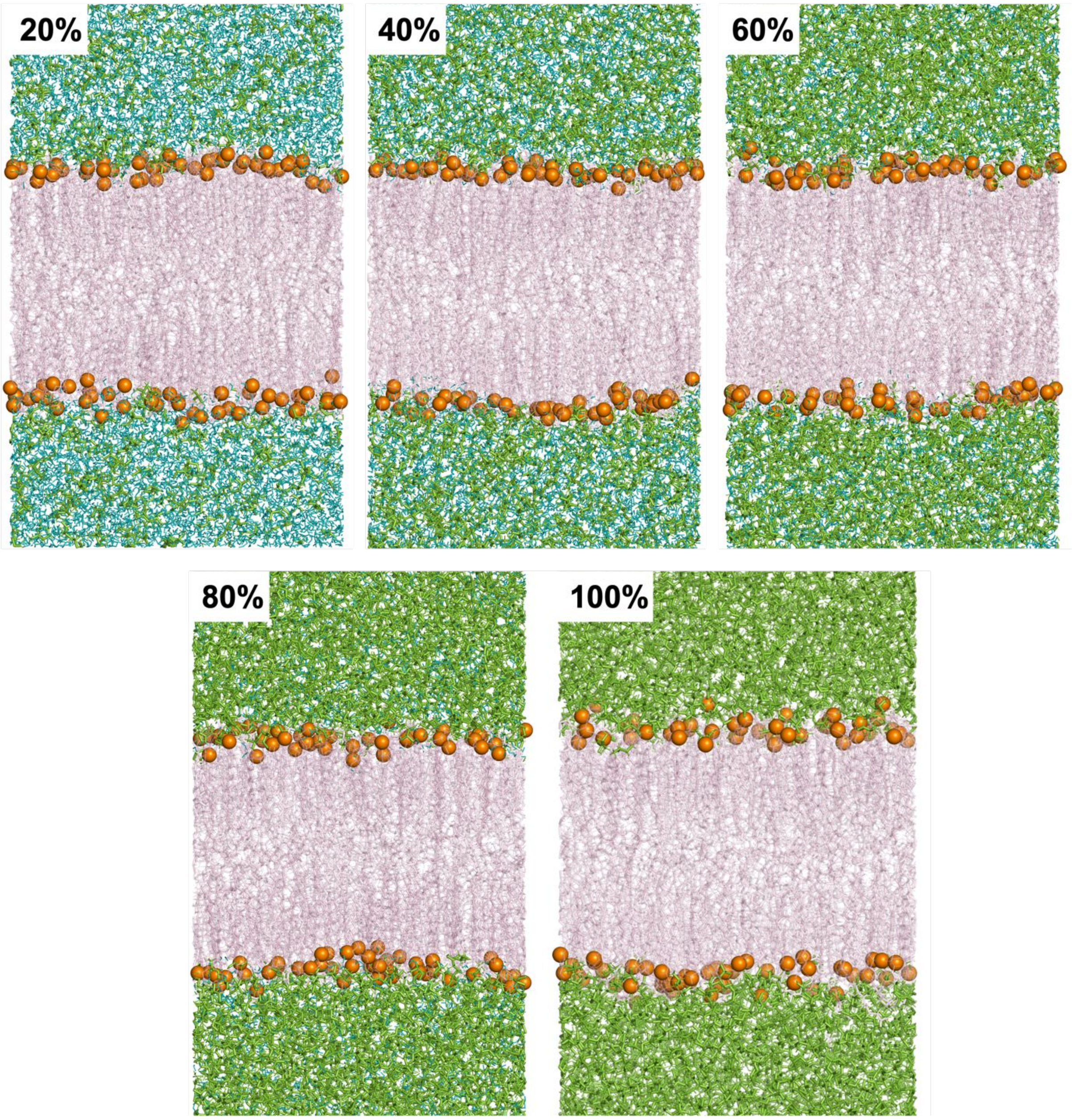

Figure S6. Snapshots taken from the last frame of the 500 ns simulations of the CHARMM bilayer solvated with 20-100% PG. The lipid tails are shown in pink, water in cyan, PG in green, and CER[NS]24 N atoms as orange spheres. All snapshots were produced using PyMol.

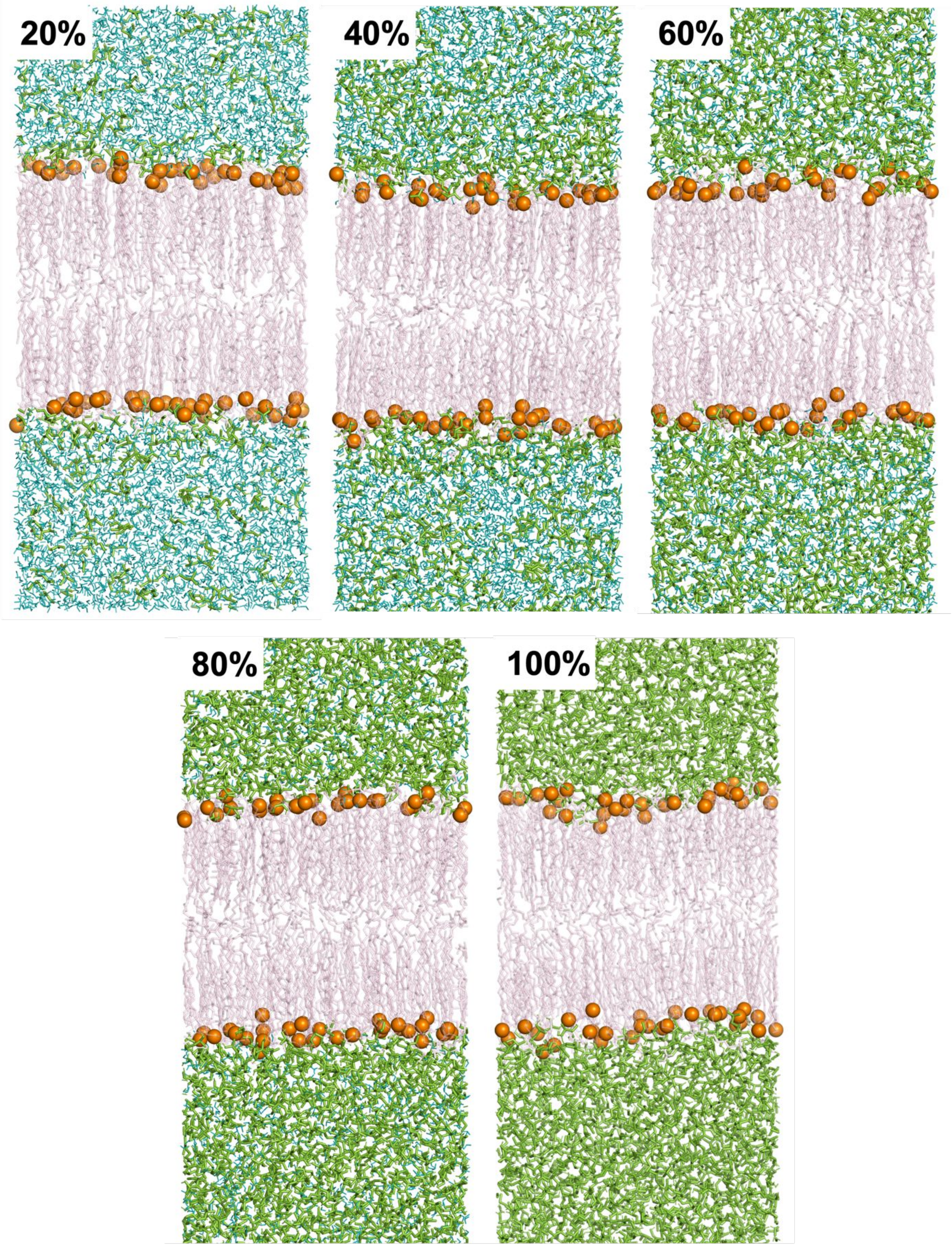

Figure S7. Snapshots taken from the last frame of the 500 ns simulations of the UA bilayer solvated with 20-100% PG. The lipid tails are shown in pink, water in cyan, PG in green, and CER[NS]24 N atoms as orange spheres.

S4 Properties of the bilayer solvated with 0-100% PG

The density profiles of each component in the CHARMM and UA systems solvated with 0-100% PG are shown in Figure S8.

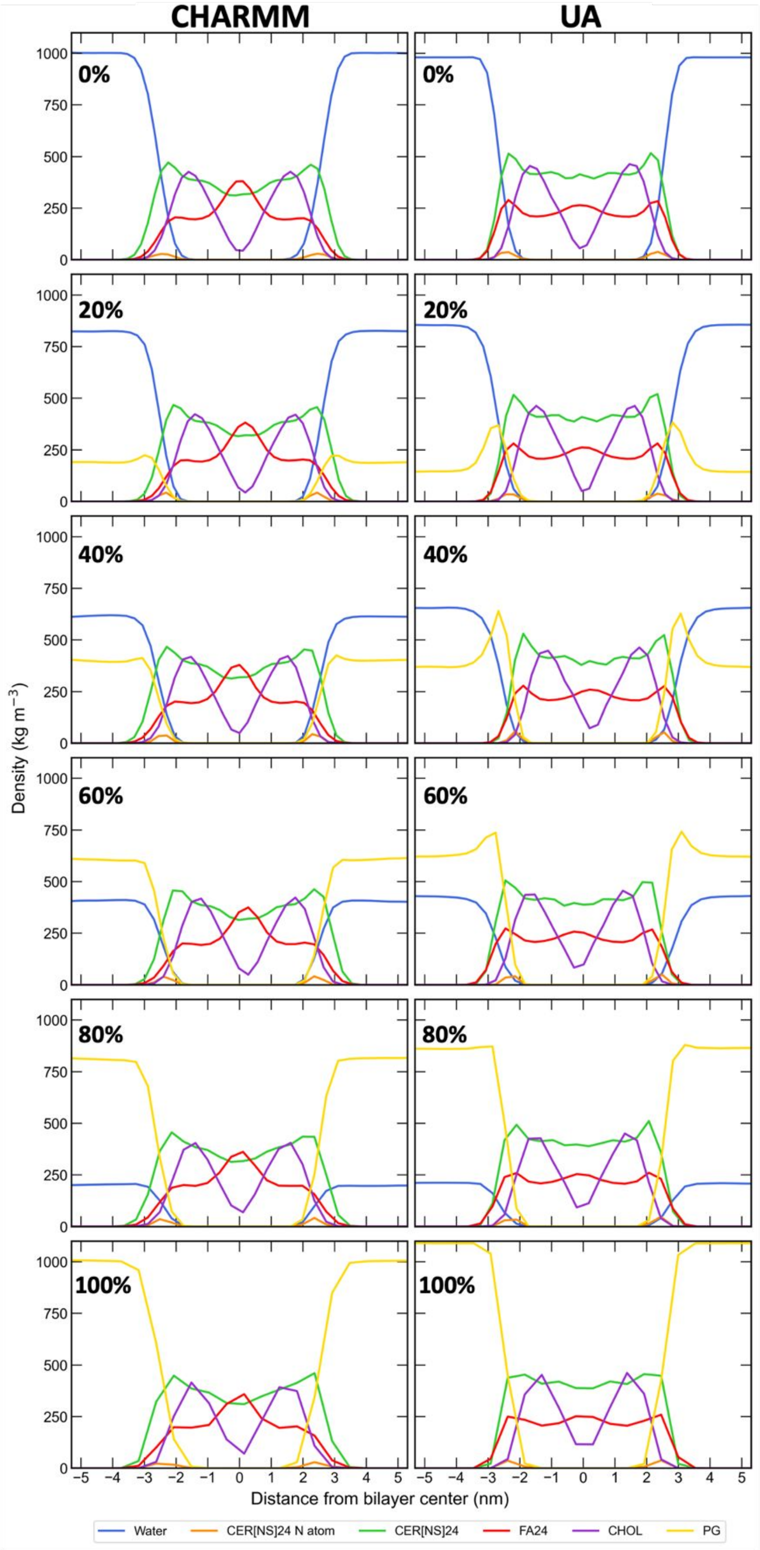

Figure S8. Density profiles for each component in the CHARMM and UA systems solvated with 0-100% PG.

The density profiles of water normalized to an effective probability density are shown in Figure S9 to allow the central region of the bilayer and the differences between the density profiles at each PG concentration to be seen more clearly. For a given PG concentration, the density profile was normalized by dividing each value by the largest value.

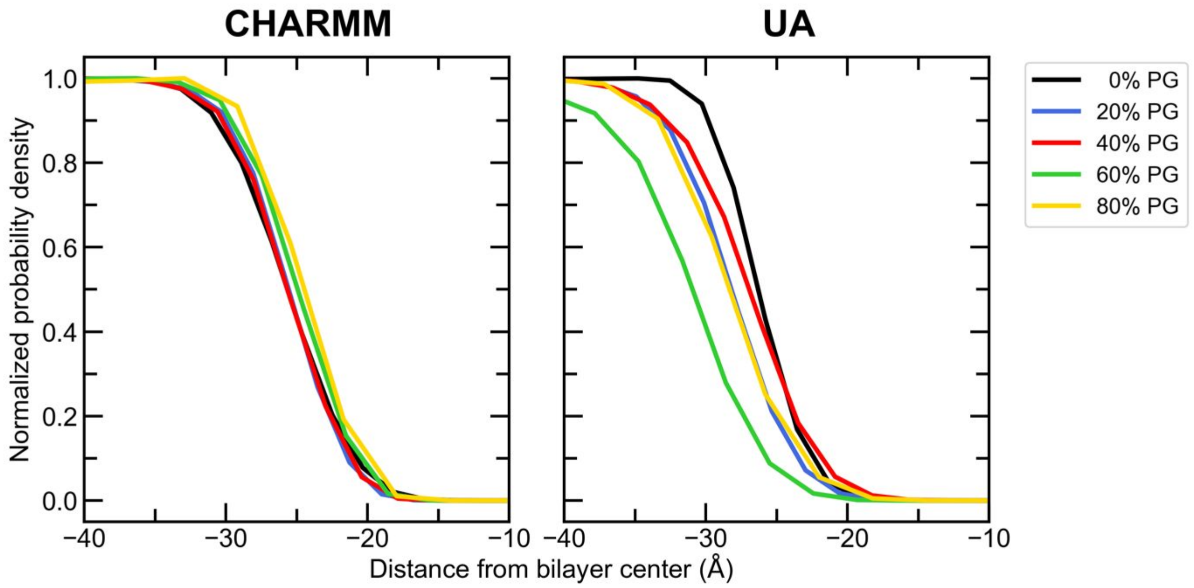

Figure S9. The density profiles of water normalized to an effective probability density for the CHARMM and UA bilayers solvated with 0-100% PG.

The number of PG-PG and PG-water H-bonds present in the CHARMM and UA systems solvated with 20-100% PG are shown in Figure S10, and the number of lipid-lipid, lipid-solvent, and solvent-solvent H-bonds present in each system are reported in Table S3.

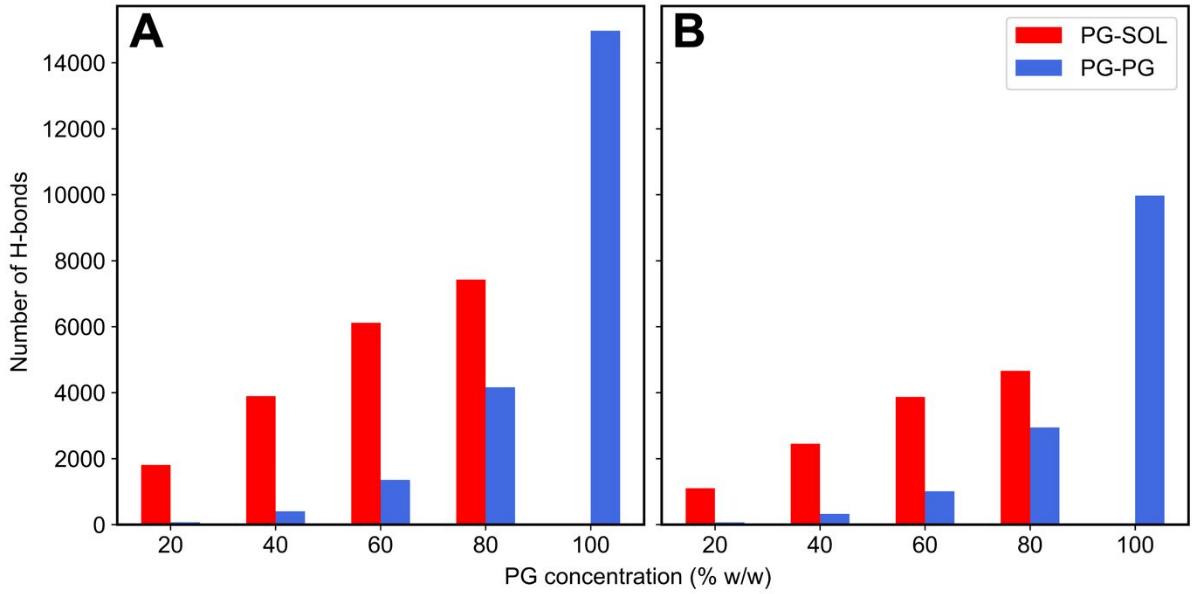

Figure S10. Number of PG-PG and PG-water H-bonds in the ***(A)*** *CHARMM and* ***(B)*** *UA systems solvated with 20-100% PG.*

Table S3. Number of lipid-lipid, lipid-solvent, and solvent-solvent H-bonds present in each system.

		Number of H-bonds													
**System**	**% PG**	**CER-CER**	**CER-CHOL**	**CER-FFA**	**CER-SOL**	**CHOL-CHOL**	**CHOL-FFA**	**CHOL-SOL**	**FFA-FFA**	**FFA-SOL**	**PG-CER**	**PG-FFA**	**PG-CHOL**	**PG-PG**	**PG-SOL**
**CHARMM**	**0**	93.4	36.7	21.3	370.8	0	4.8	162.2	1.1	165.7	-	-	-	-	-
	**20**	96.9	38.4	21.6	314.9	0	5.2	132.7	1.5	143.3	30	14	18.9	73.8	1807.8
	**40**	98.6	39.1	20.9	271.7	0	5.6	116.3	3	124.4	55.9	25	31.3	405.1	3895.2
	**60**	99.1	43.4	24	227	0	6.5	96.2	2.1	106.7	82.3	36.6	40.9	1356	6118.3
	**80**	100.6	43.5	27.8	156	0	7.7	68.2	2.5	75.1	124.5	54.7	62.8	4163.4	7430.1
	**100**	105.6	50.6	35.1	-	0	10.8	-	4.3	-	212.9	100.7	108	14971.2	-
**UA**	**0**	41	27.5	22.1	182	0	14	64.2	1	108	-	-	-	-	-
	**20**	46.5	34.8	20.4	103.1	0	14.7	29.1	1.1	73.4	61.6	28.3	25.9	68.3	1105.9
	**40**	48.8	35	20.6	74.3	0	13.6	21.9	1.2	55.8	84.1	42.8	32	323.4	2450.7
	**60**	48.9	34.2	20.4	59.8	0	14.1	17.8	1.2	44.9	99.6	51.6	36.7	1009.9	3871.8
	**80**	49.2	33.4	20.5	41.9	0	13.9	14.4	1.3	32.3	114.9	61.9	41	2948.7	4663
	**100**	50	34.4	20.4	-	0	13.8	-	1.4	-	151.3	86.5	54.1	9975.9	-

S4.1 Statistical tests

The results of the ANOVA and Tukey's HSD test for the APL, bilayer thickness and H-bonds for the CHARMM and UA systems solvated with 0-100% PG are reported in Tables S4-S11.

Table S4. Results of Tukey's HSD test for the APL of the CHARMM systems solvated with 0-100% PG.

PG concentration 1 (%)	PG concentration 2 (%)	$p < 0.05$
0	20	TRUE
0	40	TRUE
0	60	TRUE
0	80	TRUE
0	100	TRUE
20	40	TRUE
20	60	FALSE
20	80	TRUE
20	100	TRUE
40	60	TRUE
40	80	TRUE
40	100	TRUE
60	80	TRUE
60	100	TRUE
80	100	TRUE

Table S5. Results of Tukey's HSD test for the APL of the UA systems solvated with 0-100% PG.

PG concentration 1 (%)	PG concentration 2 (%)	$p < 0.05$
0	20	TRUE
0	40	TRUE
0	60	TRUE
0	80	TRUE
0	100	TRUE
20	40	TRUE
20	60	TRUE
20	80	TRUE
20	100	TRUE
40	60	TRUE
40	80	TRUE
40	100	TRUE
60	80	TRUE
60	100	TRUE
80	100	TRUE

Table S6. Results of Tukey's HSD test for the bilayer thickness of the CHARMM systems solvated with 0-100% PG.

PG concentration 1 (%)	PG concentration 2 (%)	p < 0.05
0	20	FALSE
0	40	FALSE
0	60	FALSE
0	80	FALSE
0	100	TRUE
20	40	FALSE
20	60	FALSE
20	80	FALSE
20	100	FALSE
40	60	FALSE
40	80	FALSE
40	100	FALSE
60	80	FALSE
60	100	FALSE
80	100	FALSE

Table S7. Results of Tukey's HSD test for the bilayer thickness of the UA systems solvated with 0-100% PG.

PG concentration 1 (%)	PG concentration 2 (%)	p < 0.05
0	20	FALSE
0	40	FALSE
0	60	FALSE
0	80	FALSE
0	100	TRUE
20	40	FALSE
20	60	FALSE
20	80	FALSE
20	100	TRUE
40	60	FALSE
40	80	FALSE
40	100	TRUE
60	80	FALSE
60	100	TRUE
80	100	FALSE

Table S8. p-values from the ANOVA test for CHARMM and UA lipid-lipid and lipid-solvent H-bonds.

Forcefield	H-bond type	p-value
CHARMM	LIPID-LIPID	0.999
	LIPID-WATER	0.052
	LIPID-PG	0.003
UA	LIPID-LIPID	0.999
	LIPID-WATER	0.015
	LIPID-PG	0.046

Table S9. Results of Tukey's HSD test for the lipid-PG H-bonds of the CHARMM systems solvated with 0-100% PG.

PG concentration 1 (%)	PG concentration 2 (%)	p < 0.05
0	20	FALSE
0	40	FALSE
0	60	FALSE
0	80	FALSE
0	100	TRUE
20	40	FALSE
20	60	FALSE
20	80	FALSE
20	100	TRUE
40	60	FALSE
40	80	FALSE
40	100	TRUE
60	80	FALSE
60	100	FALSE
80	100	FALSE

Table S10. Results of Tukey's HSD test for the lipid-water H-bonds of the UA systems solvated with 0-100% PG.

PG concentration 1 (%)	PG concentration 2 (%)	p < 0.05
0	20	FALSE
0	40	FALSE
0	60	FALSE
0	80	FALSE
0	100	TRUE
20	40	FALSE
20	60	FALSE
20	80	FALSE
20	100	FALSE
40	60	FALSE
40	80	FALSE
40	100	FALSE
60	80	FALSE
60	100	FALSE
80	100	FALSE

Table S11. Results of Tukey's HSD test for the lipid-PG H-bonds of the UA systems solvated with 0-100% PG.

PG concentration 1 (%)	PG concentration 2 (%)	p < 0.05
0	20	FALSE
0	40	FALSE
0	60	FALSE
0	80	FALSE
0	100	TRUE
20	40	FALSE
20	60	FALSE
20	80	FALSE
20	100	FALSE
40	60	FALSE
40	80	FALSE
40	100	FALSE
60	80	FALSE
60	100	FALSE
80	100	FALSE

S4.2 Repeat simulations

Two additional repeat simulations of the CHARMM and UA model bilayers solvated with pure water, and with 20 and 80% PG were performed. The average properties calculated from the three independent simulations of the CHARMM and UA systems containing 0, 20, and 80% PG are reported below. Error bars for the single simulation systems were obtained by block averaging over ten 40 ns blocks, and error bars for the multiple repeat systems were obtained from the standard deviation over the three independent simulations.

In the CHARMM 0% and 80% PG systems averaged over three independent simulations, the mean APL values and error bars were found to decrease compared to the results of the corresponding systems based on a single simulation, while the APL and error bars were found to increase for the 20% PG system based on three independent simulations (Figure S11). For the UA systems averaged over three independent simulations, the error bars were larger than the corresponding single-simulation systems (Figure S12).

In the UA systems averaged over three independent simulations, the mean bilayer thickness values and error bars were found to decrease compared to the results of the corresponding systems based on a single simulation (Figure S12). The mean bilayer thickness values of the CHARMM systems averaged over three independent simulations were found to increase slightly, and the error bars were found to decrease (Figure S11).

The density profiles of each component in the CHARMM bilayer solvated with 0, 20, and 80% PG averaged over three independent simulations are shown in Figures S13-15, and the density profiles of each component in the UA bilayer solvated with 0, 20, and 80% PG averaged over three independent simulations are shown in Figures S16-18. The lipid tail order parameters in the CHARMM bilayer solvated with 0, 20, and 80% PG averaged over three independent simulations are shown in Figure S19, and the lipid tail order parameters in the UA bilayer solvated with 0, 20, and 80% PG averaged over three independent simulations are shown in Figure S20.

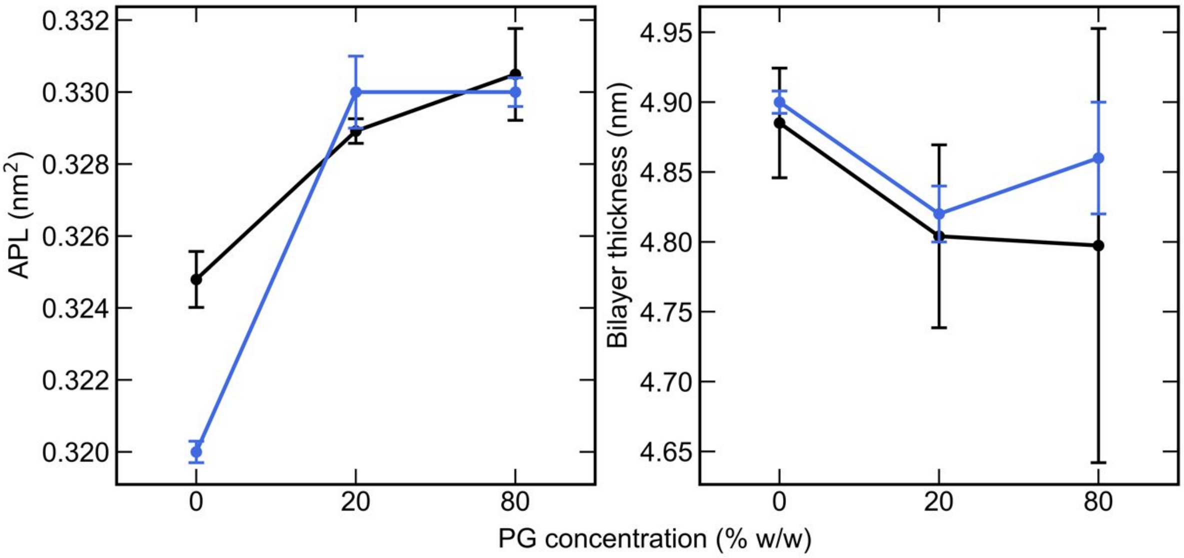

Figure S11. The APL and bilayer thickness of the CHARMM 0, 20, and 80% PG systems averaged over three independent simulations (blue), and the corresponding values obtained from the systems based on a single simulation (black).

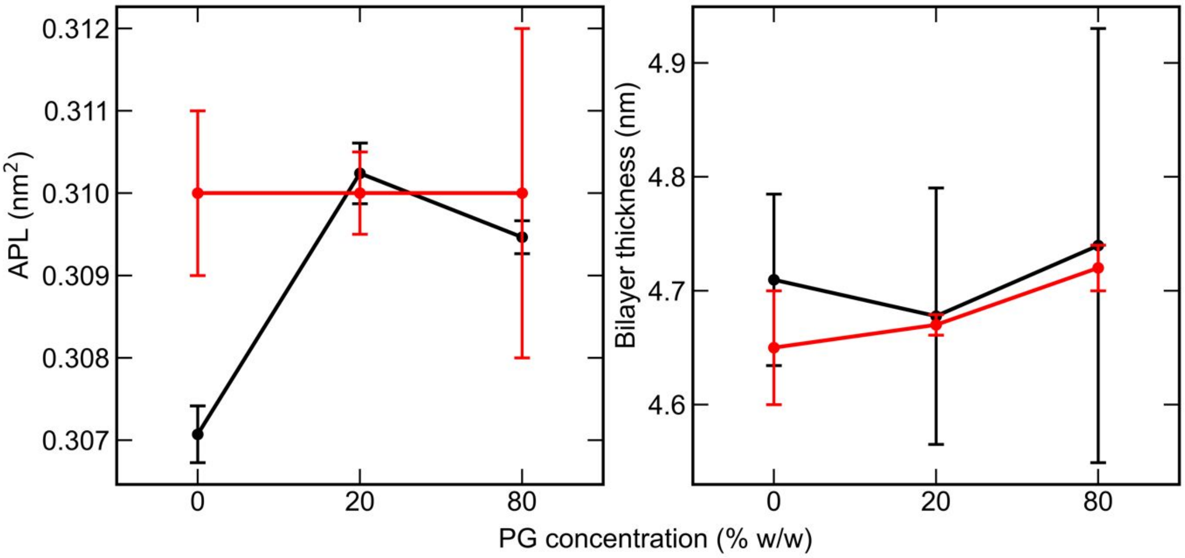

Figure S12. The APL and bilayer thickness of the UA 0, 20, and 80% PG systems averaged over three independent simulations (red), and the corresponding values obtained from the systems based on a single simulation (black).

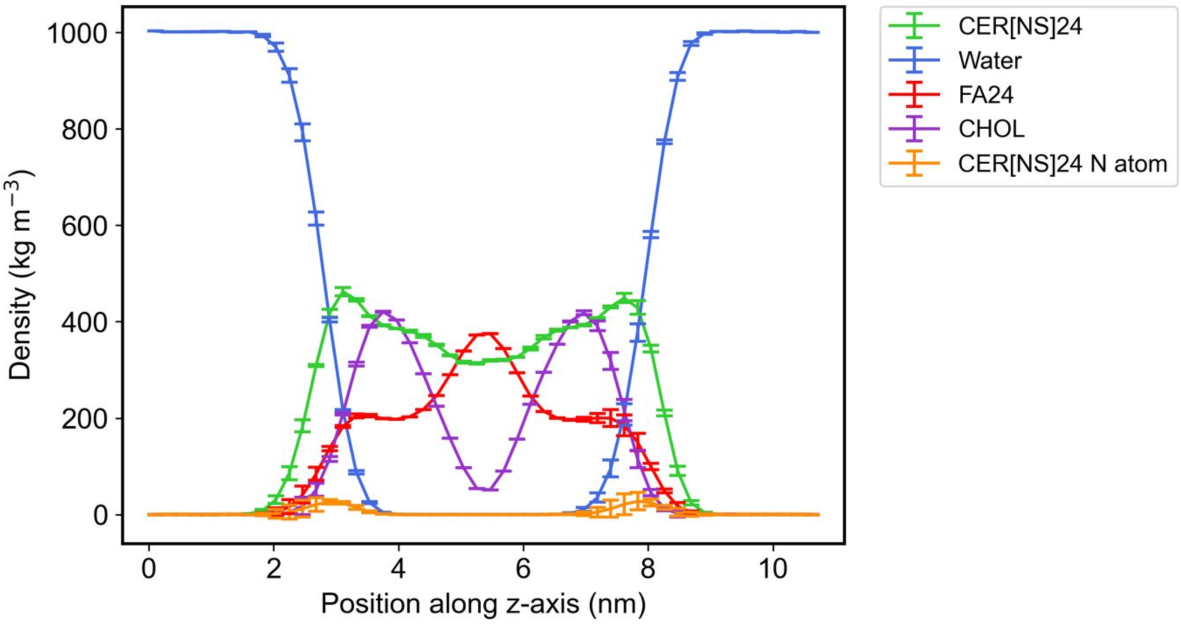

Figure S13. Density profile of each component in the CHARMM bilayer solvated with pure water, averaged over three independent simulations.

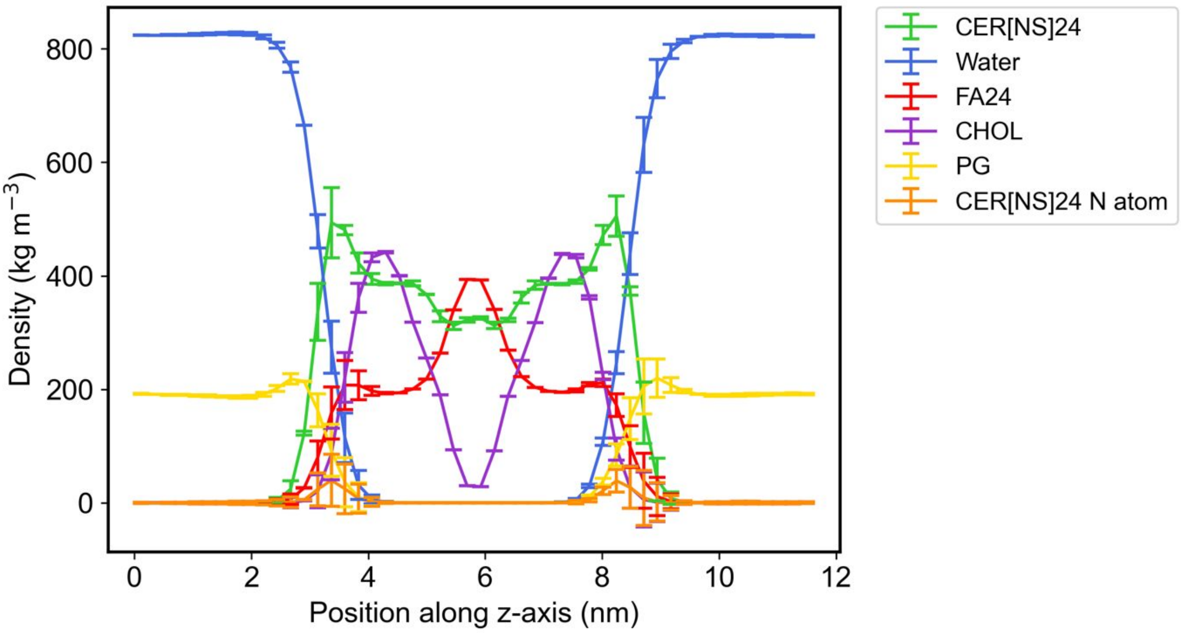

Figure S14. Density profile of each component in the CHARMM bilayer solvated with 20% PG, averaged over three independent simulations.

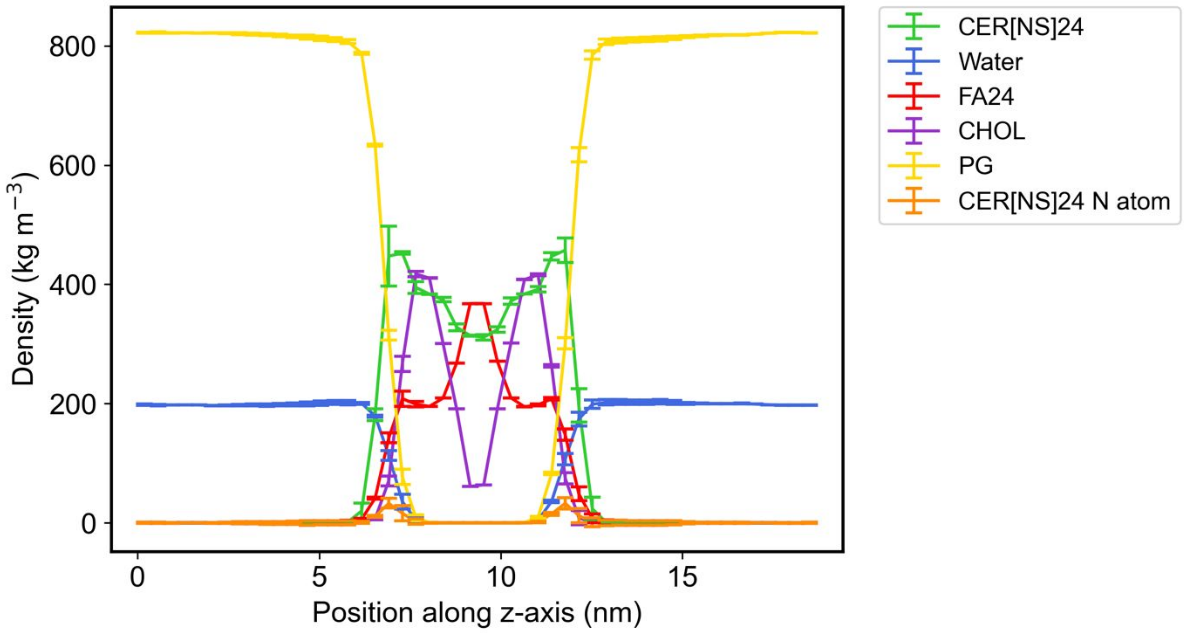

Figure S15. Density profile of each component in the CHARMM bilayer solvated with 80% PG, averaged over three independent simulations.

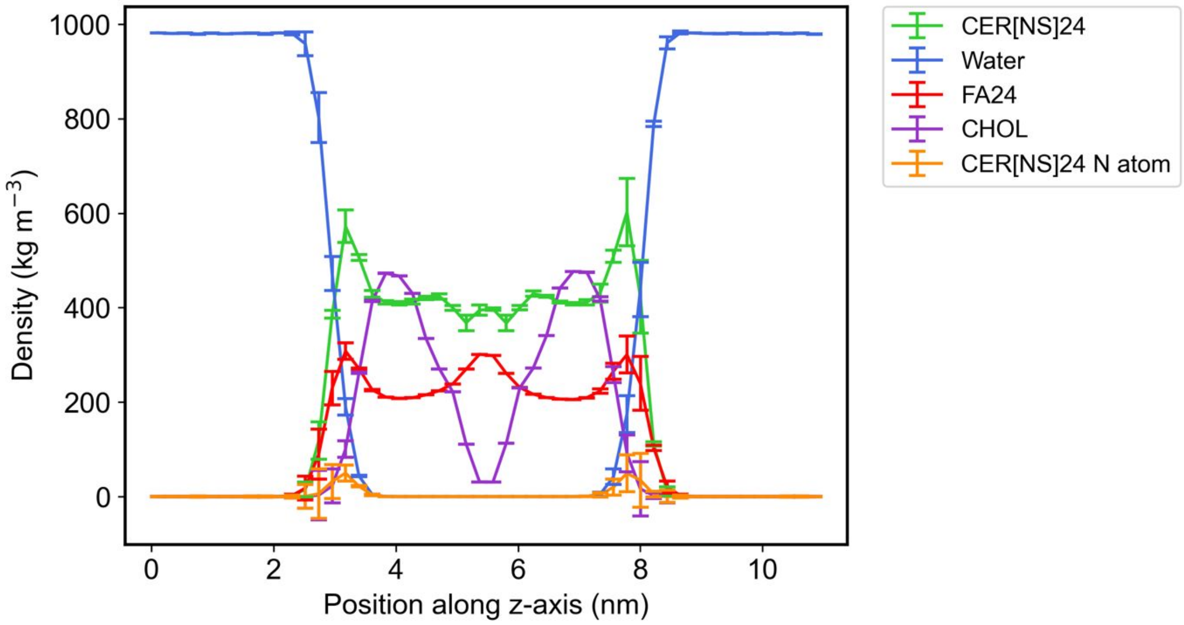

Figure S16. Density profile of each component in the UA bilayer solvated with pure water, averaged over three independent simulations.

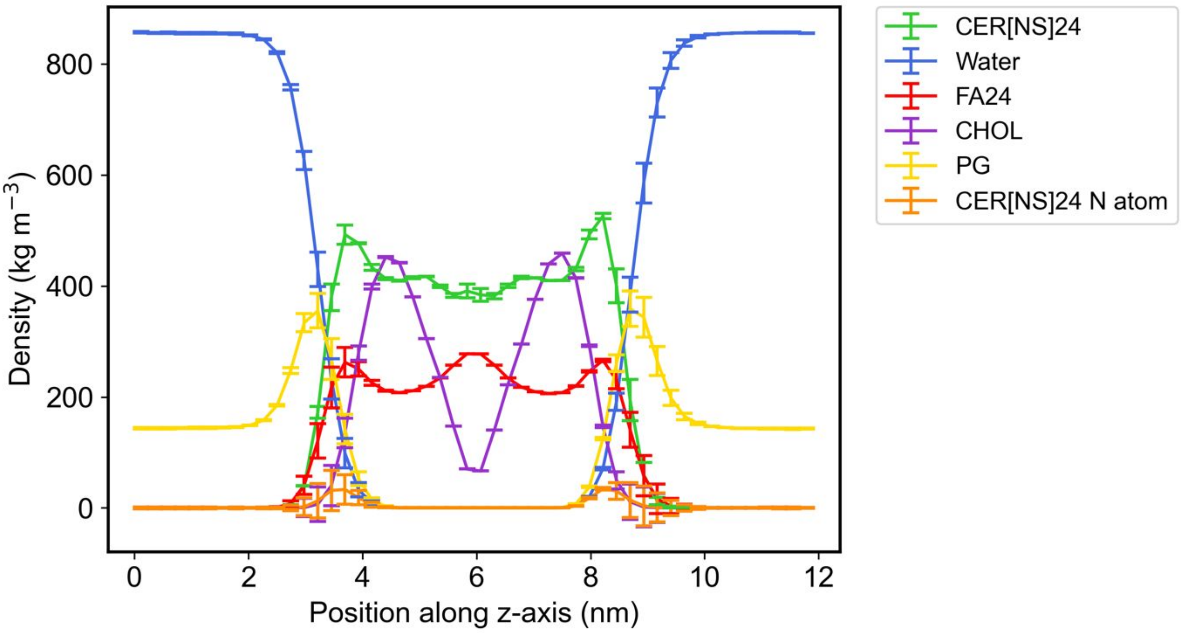

Figure S17. Density profile of each component in the UA bilayer solvated with 20% PG, averaged over three independent simulations.

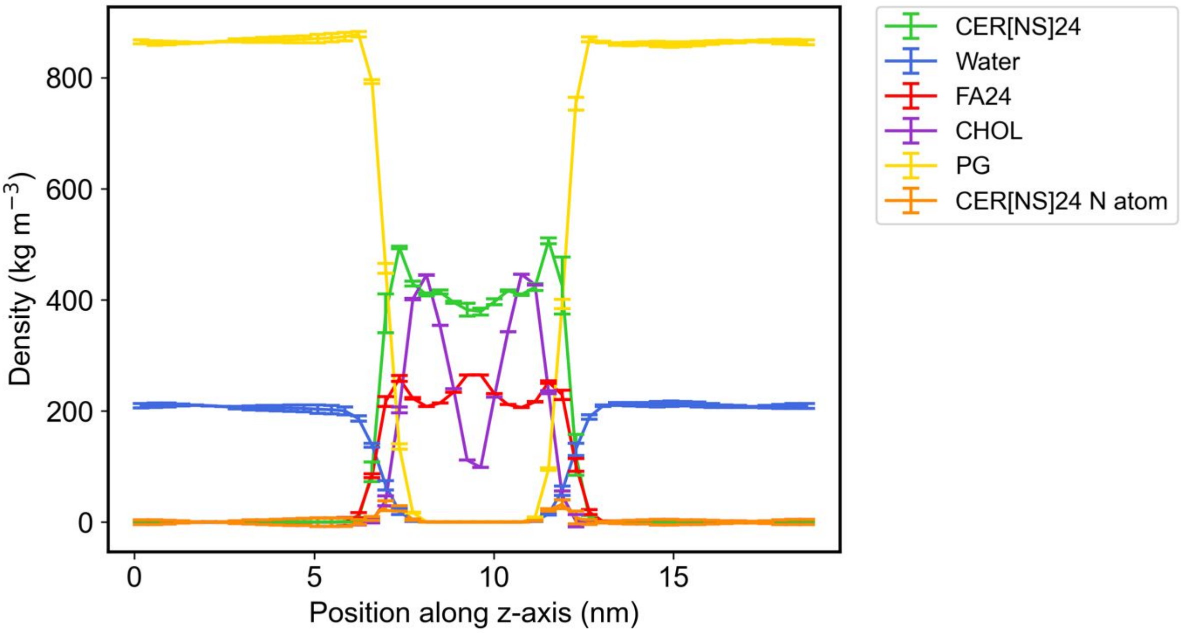

Figure S18. Density profile of each component in the UA bilayer solvated with 80% PG, averaged over three independent simulations.

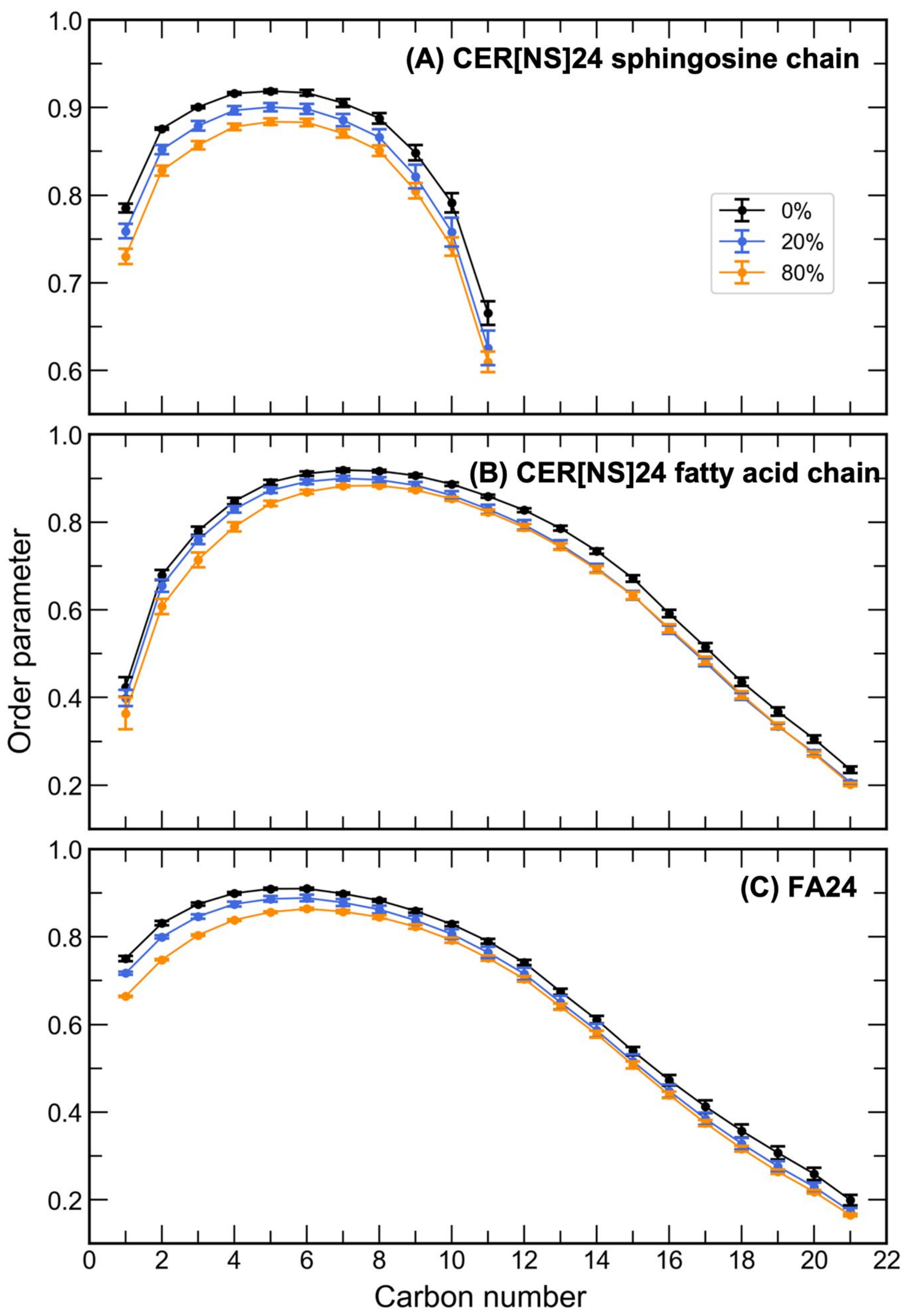

Figure S19. Lipid-tail order parameters for the ***(A)*** *sphingosine and* ***(B)*** *fatty acid chains of CER[NS]24, and* ***(C)*** *FA24 in the CHARMM bilayers solvated with 0, 20, and 80% PG, averaged over three independent simulations.*

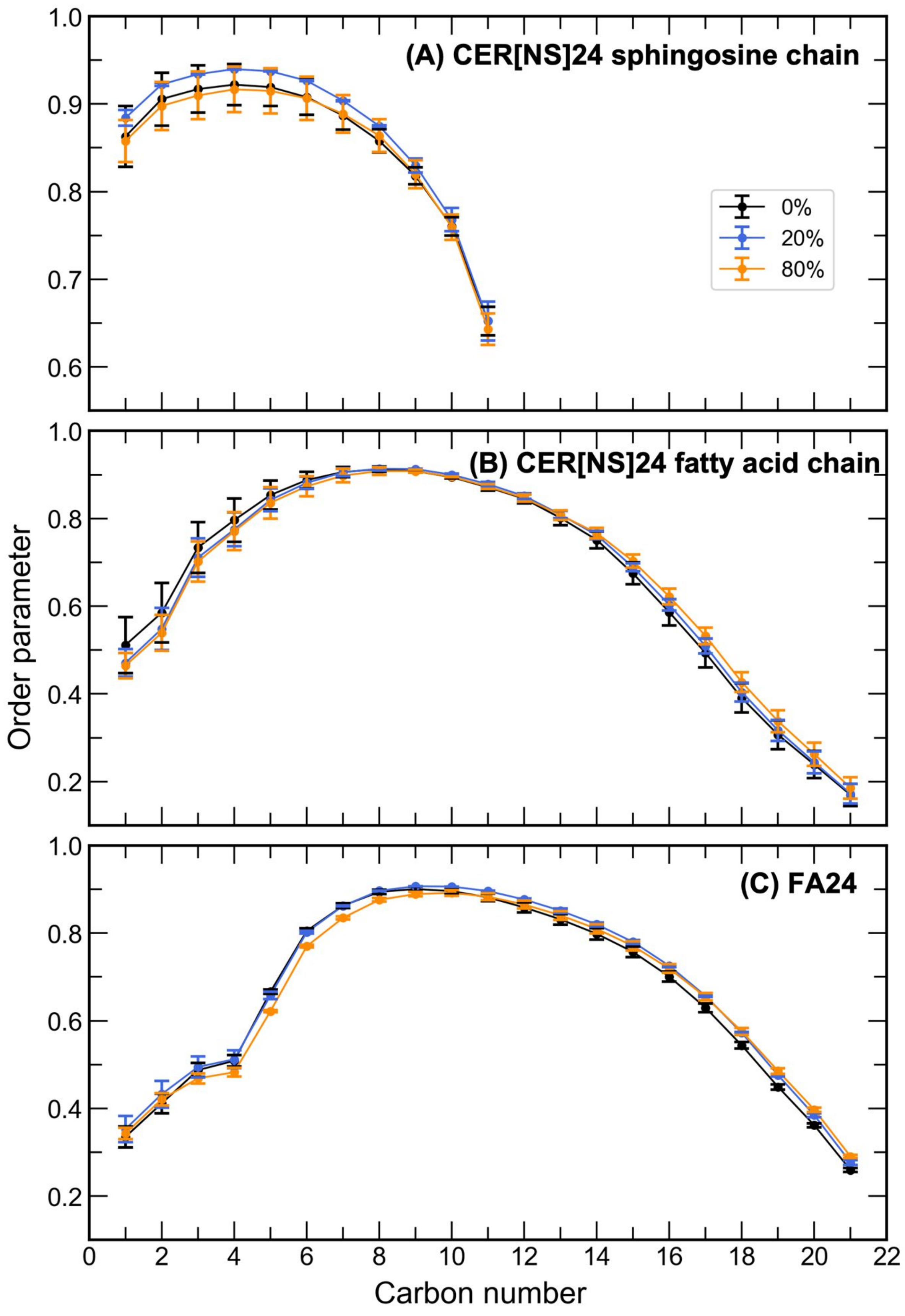

Figure S20. Lipid-tail order parameters for the ***(A)*** *sphingosine and* ***(B)*** *fatty acid chains of CER[NS]24, and* ***(C)*** *FA24 in the UA bilayers solvated with 0, 20, and 80% PG, averaged over three independent simulations.*

S5 CHARMM CER[NP]24/FA24/CHOL bilayer simulations

An additional CHARMM bilayer composed of CER[NP]24 (also referred to as CER3), FA24, and CHOL (1:1:1) was simulated and solvated with 80% PG. A snapshot from the last frame of the simulation of 80% PG with the CER[NP]24/FA24/CHOL bilayer is shown in Figure S21A. As was seen for the CHARMM CER[NS]24/FA24/CHOL bilayer solvated with 80% PG, PG preferred to localise in the lipid headgroup regions, rather than permeate the bilayer. This is evident from the system density profiles shown in Figure S21B. The structure of CER[NP]24 is given in Figure S21C.

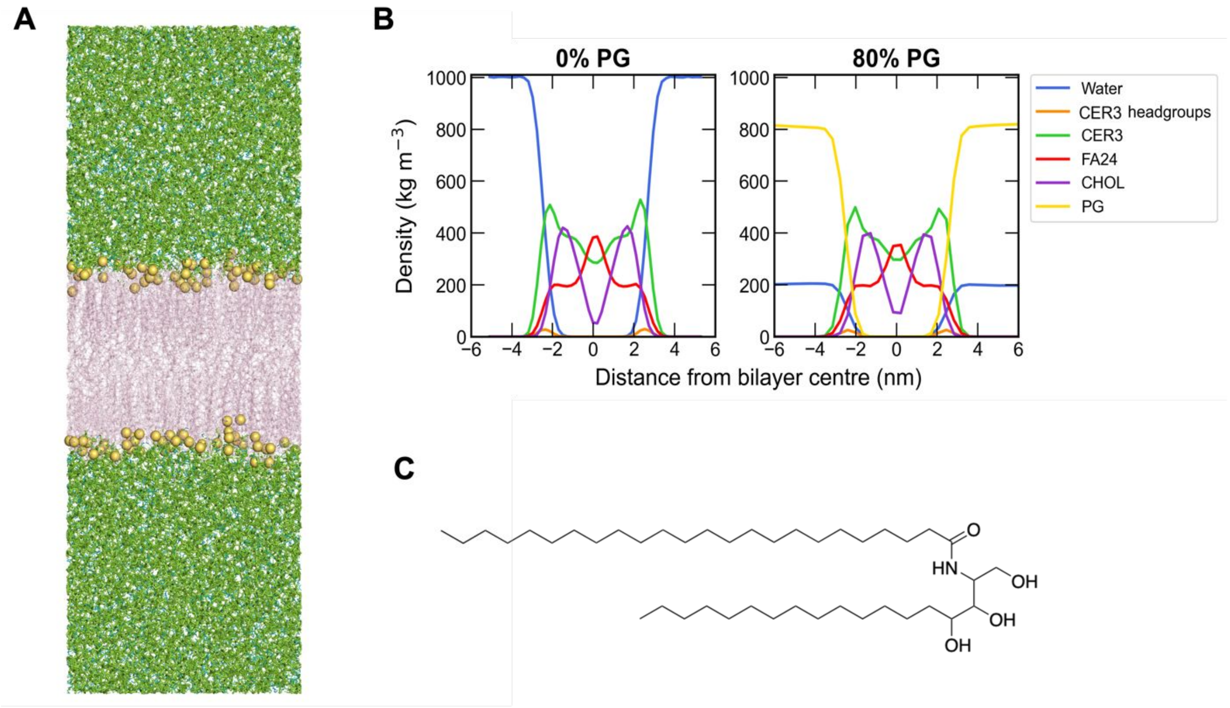

Figure S21. ***(A)*** *Snapshot taken from the last frame of the 500 ns simulation of the CHARMM CER[NP]24/FA24/CHOL bilayer solvated with 80% PG. Lipids are shown in pink, CER[NP]24 N atoms as yellow spheres, PG in green, and water in cyan.* ***(B)*** *Density profiles for each component of the CHARMM CER[NP]24/FA24/CHOL bilayer in water and in 80% PG.* ***(C)*** *Structure of CER[NP]24.*

S6 Extended 80% PG simulations

Snapshots taken from the last frame of simulations of the UA and CHARMM bilayers solvated with 80% PG that were extended to 2 μs are shown in Figures S22 and S23 respectively.

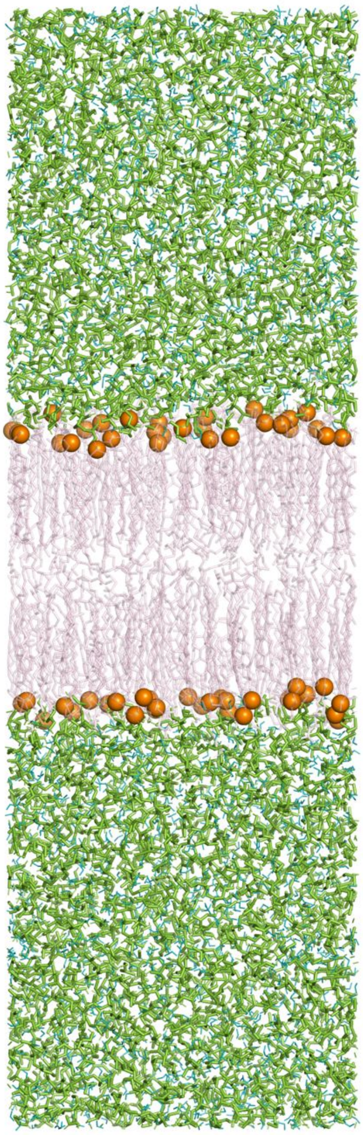

Figure S22. Snapshot taken from the last frame of the 2 μs simulation of the UA CER[NS]24/FA24/CHOL bilayer solvated with 80% PG. Lipids are shown in pink, CER[NS]24 N atoms as orange spheres, PG in green, and water in cyan.

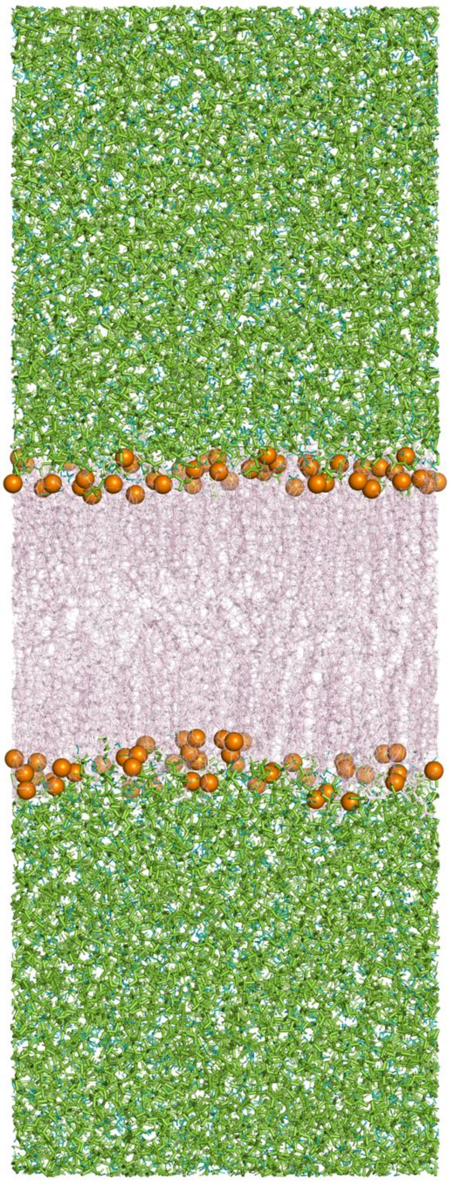

Figure S23. Snapshot taken from the last frame of the 2 µs simulation of the CHARMM CER[NS]24/FA24/CHOL bilayer solvated with 80% PG. Lipids are shown in pink, CER[NS]24 N atoms as orange spheres, PG in green, and water in cyan.

S7 Simulations with PG starting inside the bilayer

To determine whether the lack of spontaneous permeation was due to our initial configurations (with the PG in the aqueous phase) we carried out an additional simulation of the bilayer with 46 PG molecules inserted into the interior of the CHARMM membrane solvated with pure water. The system was simulated for 500 ns, using the same simulation parameters as those used for the CHARMM bilayer solvated with PG (see Section 2.5 of the main paper). The starting configuration for this system was generated using PACKMOL to insert PG molecules into spaces in the central region of the bilayer.

Selected snapshots from the trajectory of the resultant simulation are shown in Figure S24. It can be seen that within the first 80 ns, the PG molecules aggregate into a single cluster that then diffuses laterally within the membrane interior. After ~250 ns, some of the PG molecules started to move towards the upper leaflet, and 4 PG molecules exited the bilayer. The remaining PG molecules re-grouped to form a cluster, and then at ~390 ns the cluster of PG molecules started to move towards the upper leaflet again. By 406 ns all PG molecules had exited the bilayer. Once the molecules had exited bilayer, they remained in the solvent phase for the rest of the simulation and did not re-enter the bilayer.

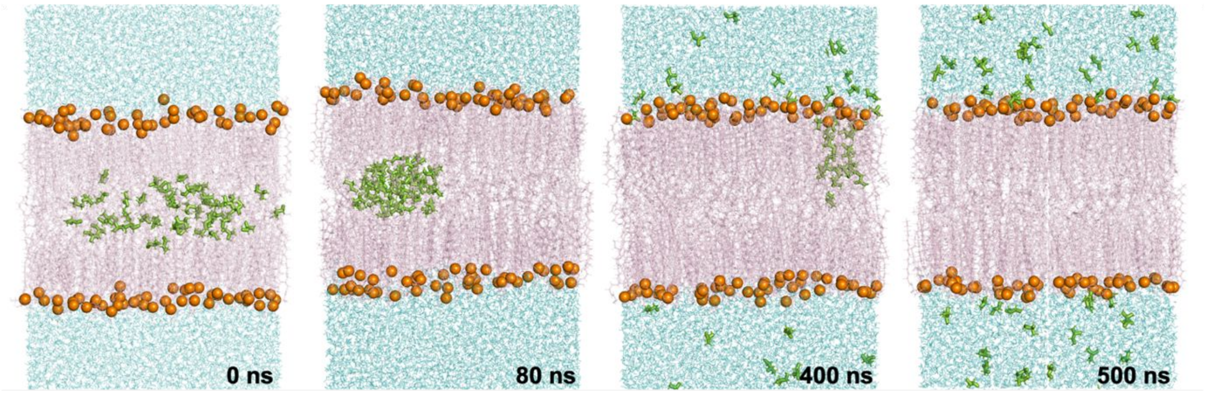

Figure S24. Snapshots taken from key points in the 500 ns simulation of 46 PG starting inside the CHARMM bilayer. The lipid tails are shown in pink, water in cyan, PG in green, and CER[NS]24 N atoms as orange spheres.

References

1. Ferreira, E.S.C., et al., *Computational and experimental study of propeline: A choline chloride based deep eutectic solvent.* Journal of Molecular Liquids, 2020. **298**: p. 111978.
2. Ferreira, E.S.C., et al., *New Force Field Model for Propylene Glycol: Insight to Local Structure and Dynamics.* J Phys Chem B, 2017. **121**(48): p. 10906-10921.
