# Supplementary material for: Mechanisms of the Drug Penetration Enhancer Propylene Glycol Interacting with Skin Lipid Membranes
Source: J Phys Chem B. 2024 Apr 16;128(16):3885–97. doi: 10.1021/acs.jpcb.3c06784 (PMC11056976; doi:10.1021/acs.jpcb.3c06784)
Supplement: Supplementary file 2 — jp3c06784_si_002.pdf [file jp3c06784_si_002.pdf]

```
;----------------------------TITLE -----------------------------------------------------------------------------------------
;   (S)-(+)-1,2-Propanediol
;
; This file was generated at 00:21 on 2021-12-18 by
;
;                  Automatic Topology Builder
;
;                   REVISION 2021-05-20 12:41:00
;----------------------------------------------------------------------------------------------------------------------------
; Authors     : Martin Stroet, Bertrand Caron, Alpeshkumar K. Malde, Thomas Lee, Alan E. Mark
;
; Institute   : Molecular Dynamics group,
;               School of Chemistry and Molecular Biosciences (SCMB),
;               The University of Queensland, QLD 4072, Australia
; URL         : https://atb.uq.edu.au
; Citations   : 1. Malde AK, Zuo L, Breeze M, Stroet M, Poger D, Nair PC, Oostenbrink C, Mark AE.
;                  An Automated force field Topology Builder (ATB) and repository: version 1.0.
;                  Journal of Chemical Theory and Computation, 2011, 7, 4026-4037.
;               2. Stroet M, Caron B, Visscher K, Geerke D, Malde AK, Mark AE.
;                  Automated Topology Builder version 3.0: Prediction of solvation free enthalpies in water and hexane.
;                  DOI:10.1021/acs.jctc.8b00768
;
; Disclaimer  :
;      While every effort has been made to ensure the accuracy and validity of parameters provided below
;      the assignment of parameters is being based on an automated procedure combining data provided by a
;      given user as well as calculations performed using third party software. They are provided as a guide.
;      The authors of the ATB cannot guarantee that the parameters are complete or that the parameters provided
;      are appropriate for use in any specific application. Users are advised to treat these parameters with discretion
;      and to perform additional validation tests for their specific application if required. Neither the authors
;      of the ATB or The University of Queensland except any responsibly for how the parameters may be used.
;
; Release notes and warnings:
;  (1) The topology is based on a set of atomic coordinates and other data provided by the user after
;      after quantum mechanical optimization of the structure using different levels of theory depending on
;      the nature of the molecule.
;  (2) In some cases the automatic bond, bond angle and dihedral type assignment is ambiguous.
;      In these cases alternative type codes are provided at the end of the line.
;  (3) While bonded parameters are taken where possible from the nominated force field non-standard bond, angle and dihedral
;      type code may be incorporated in cases where an exact match could not be found. These are marked as "non-standard"
;      or "uncertain" in comments.
;  (4) In some cases it is not possible to assign an appropriate parameter automatically. "%%" is used as a place holder
;      for those fields that could not be determined automatically. The parameters in these fields must be assigned manually
;      before the file can be used.
;----------------------------------------------------------------------------------------------------------------------------
; Input Structure : PGO
; Output          : UNITED ATOM topology
;       Use in conjunction with the corresponding united atom PDB file.
;----------------------------------------------------------------------------------------------------------------------------
; Citing this topology file
; ATB molid: 9692
; ATB Topology Hash: f0578
;----------------------------------------------------------------------------------------------------------------------------
; Final Topology Generation was performed using:
; A B3LYP/6-31G* optimized geometry.
; Bonded and van der Waals parameters were taken from the GROMOS 54A7 parameter set.
; Initial charges were estimated using the ESP method of Merz-Kollman.
; Final charges and charge groups were generated by method described in the ATB paper.
; If required, additional bonded parameters were generated from a Hessian matrix calculated at the B3LYP/6-31G* level of theory.
;----------------------------------------------------------------------------------------------------------------------------
;
;
[ moleculetype ]
; Name   nrexcl
PGO      3
[ atoms ]
;  nr  type  resnr  resid  atom  cgnr  charge    mass
    1  HS14    1     PGO     H8    1    0.373   1.0080
    2  OAlc    1     PGO     O2    2   -0.628  15.9994
    3  CPos    1     PGO     C2    3    0.267  12.0110
    4  ACH3    1     PGO     C3    5   -0.016  15.0350          ; changed CH3 to ACH3
    5  ACH2    1     PGO     C1    6    0.224  14.0270
    6  OAlc    1     PGO     O1    7   -0.619  15.9994
    7     H    1     PGO     H7    8    0.399   1.0080
; total charge of the molecule:   0.000
[ bonds ]
;  ai   aj  funct   c0         c1
    1    2    2   0.0972   1.9581e+07
    2    3    2   0.1430   8.1800e+06
    3    4    2   0.1530   7.1500e+06
    3    5    2   0.1530   7.1500e+06
    5    6    2   0.1430   8.1800e+06
    6    7    2   0.0972   1.9581e+07
[ pairs ]
;  ai   aj  funct  ;  all 1-4 pairs but the ones excluded in GROMOS itp
    1    4    1
    1    5    1
    2    6    1
    3    7    1
    4    6    1
[ angles ]
;  ai   aj   ak  funct   angle     fc
    1    2    3    2    109.50   450.00
    2    3    4    2    111.00   530.00
    2    3    5    2    109.50   520.00
    4    3    5    2    111.00   530.00
    3    5    6    2    111.00   530.00
    5    6    7    2    109.50   450.00
[ dihedrals ]
; GROMOS improper dihedrals
;  ai   aj   ak   al  funct   angle     fc
    3    5    4    2    2     35.26   334.72
[ dihedrals ]
;  ai   aj   ak   al  funct    ph0      cp     mult
    1    2    3    5    1      0.00     1.26    3
    3    5    6    7    1      0.00     1.26    3
    4    3    5    6    1      0.00     5.92    3
[ exclusions ]
;  ai   aj  funct  ;  GROMOS 1-4 exclusions
```
