# Supplementary material for: Mechanisms of the Drug Penetration Enhancer Propylene Glycol Interacting with Skin Lipid Membranes
Source: J Phys Chem B. 2024 Apr 16;128(16):3885–97. doi: 10.1021/acs.jpcb.3c06784 (PMC11056976; doi:10.1021/acs.jpcb.3c06784)
Supplement: Supplementary file 3 — jp3c06784_si_003.pdf [file jp3c06784_si_003.pdf]

```
; Created by cgenff_charmm2gmx.py

[ moleculetype ]
; Name                  nrexcl
PGO                          3

[ atoms ]
;        nr              type  resnr residue  atom   cgnr         charge              mass  typeB
chargeB         massB
; residue        1 PGO rtp PGO q        qsum
     1      CG331      1    PGO     C1      1     -0.266     12.011   ;
     2      CG311      1    PGO     C2      2      0.122     12.011   ;
     3      CG321      1    PGO     C3      3      0.062     12.011   ;
     4      OG311      1    PGO     O4      4     -0.649     15.999   ;
     5      OG311      1    PGO     O5      5     -0.647     15.999   ;
     6       HGP1      1    PGO     H6      6      0.419      1.008   ;
     7       HGA3      1    PGO     H2      7      0.090      1.008   ;
     8       HGA3      1    PGO     H3      8      0.090      1.008   ;
     9       HGA3      1    PGO     H4      9      0.090      1.008   ;
    10       HGA1      1    PGO     H5     10      0.090      1.008   ;
    11       HGA2      1    PGO     H1     11      0.090      1.008   ;
    12       HGA2      1    PGO     H7     12      0.090      1.008   ;
    13       HGP1      1    PGO     H8     13      0.419      1.008   ;

[ bonds ]
;       ai        aj funct                        c0                    c1                          c2
                       c3
    1     2      1 ;       CG331      CG311
    1     7      1 ;       CG331       HGA3
    1     8      1 ;       CG331       HGA3
    1     9      1 ;       CG331       HGA3
    2     3      1 ;       CG311      CG321
    2     5      1 ;       CG311      OG311
    2    10      1 ;       CG311       HGA1
    3     4      1 ;       CG321      OG311
    3    11      1 ;       CG321       HGA2
    3    12      1 ;       CG321       HGA2
    4    13      1 ;       OG311       HGP1
    5     6      1 ;       OG311       HGP1

[ pairs ]
;       ai        aj funct                        c0                    c1                          c2
                       c3
    1     4      1
    1    11      1
    1    12      1
    1     6      1
    2    13      1
    3     7      1
    3     8      1
    3     9      1
    3     6      1
    4     5      1
    4    10      1
    5     7      1
    5     8      1
    5     9      1
    5    11      1
    5    12      1
    6    10      1
    7    10      1
    8    10      1
    9    10      1
   10    11      1
   10    12      1
   11    13      1
   12    13      1

[ angles ]
;       ai        aj    ak funct                        c0                          c1
        c2                       c3
    2     1     7       5 ;      CG311      CG331       HGA3
    2     1     8       5 ;      CG311      CG331       HGA3
    2     1     9       5 ;      CG311      CG331       HGA3
    7     1     8       5 ;       HGA3      CG331       HGA3
    7     1     9       5 ;       HGA3      CG331       HGA3
    8     1     9       5 ;       HGA3      CG331       HGA3
    1     2     3       5 ;      CG331      CG311      CG321
    1     2     5       5 ;      CG331      CG311      OG311
    1     2    10       5 ;      CG331      CG311       HGA1
    3     2     5       5 ;      CG321      CG311      OG311
```

```
    3     2    10       5 ;      CG321      CG311       HGA1
    5     2    10       5 ;      OG311      CG311       HGA1
    2     3     4       5 ;      CG311      CG321      OG311
    2     3    11       5 ;      CG311      CG321       HGA2
    2     3    12       5 ;      CG311      CG321       HGA2
    4     3    11       5 ;      OG311      CG321       HGA2
    4     3    12       5 ;      OG311      CG321       HGA2
   11     3    12       5 ;       HGA2      CG321       HGA2
    3     4    13       5 ;      CG321      OG311       HGP1
    2     5     6       5 ;      CG311      OG311       HGP1

[ dihedrals ]
;       ai        aj    ak        al funct                       c0                    c1
                  c2                   c3                        c4                    c5
    7     1     2     3  9 ;       HGA3      CG331      CG311      CG321
    7     1     2     5  9 ;       HGA3      CG331      CG311      OG311
    7     1     2    10  9 ;       HGA3      CG331      CG311       HGA1
    8     1     2     3  9 ;       HGA3      CG331      CG311      CG321
    8     1     2     5  9 ;       HGA3      CG331      CG311      OG311
    8     1     2    10  9 ;       HGA3      CG331      CG311       HGA1
    9     1     2     3  9 ;       HGA3      CG331      CG311      CG321
    9     1     2     5  9 ;       HGA3      CG331      CG311      OG311
    9     1     2    10  9 ;       HGA3      CG331      CG311       HGA1
    1     2     3     4  9 ;      CG331      CG311      CG321      OG311
    1     2     3    11  9 ;      CG331      CG311      CG321       HGA2
    1     2     3    12  9 ;      CG331      CG311      CG321       HGA2
    5     2     3     4  9 ;      OG311      CG311      CG321      OG311
    5     2     3    11  9 ;      OG311      CG311      CG321       HGA2
    5     2     3    12  9 ;      OG311      CG311      CG321       HGA2
   10     2     3     4  9 ;       HGA1      CG311      CG321      OG311
   10     2     3    11  9 ;       HGA1      CG311      CG321       HGA2
   10     2     3    12  9 ;       HGA1      CG311      CG321       HGA2
    1     2     5     6  9 ;      CG331      CG311      OG311       HGP1
    3     2     5     6  9 ;      CG321      CG311      OG311       HGP1
   10     2     5     6  9 ;       HGA1      CG311      OG311       HGP1
    2     3     4    13  9 ;      CG311      CG321      OG311       HGP1
   11     3     4    13  9 ;       HGA2      CG321      OG311       HGP1
   12     3     4    13  9 ;       HGA2      CG321      OG311       HGP1
```
